# Moxibustion Treatment, Alongside Conventional Western and Chinese Herbal Medical Therapies, May Improve Survival in Stage-IV Pulmonary Adenocarcinomas in a Dosage-Dependent Manner: A Prospective Observational Study With Propensity Score Analysis

**DOI:** 10.1177/15347354251342739

**Published:** 2025-06-19

**Authors:** Hegen Li, Veronika Lindberg, Lihua Zhu, Xiange Huang, Jiali Feng, Jan P. A. Baak

**Affiliations:** 1 Longhua University Hospital, Shanghai, China; 2 Lintech AS, Kristiansand, Norway; 3 Stavanger University Hospital, Norway

**Keywords:** NSCLC, adenocarcinoma, stage IV, chemotherapy, target therapy, moxibustion, prognosis

## Abstract

**Background:**

25% to 30% of primary stage IV pulmonary adenocarcinomas (PUAD-IV) die within 3 months. Many ≥3 months survivors at long follow-up are alive with disease (AWD). Platinum-based chemotherapy (PBC), tyrosine kinase inhibitors- targeted therapy (TKI-TT), and Chinese herbal medicines (oral CHM) improve prognosis. In China, moxibustion treatment (Moxa) is also used, without prognostic proof.

**Methods:**

Prospective observational Moxa evaluation in 412 first-onset consecutive PUAD-IV performance score 0 to 1 patients with 3 to 120 months follow-up. All received oral CHM with PBC, TKI-TT, or PBC + TKI-TT. Moxa was given as indicated at the start of the treatment (and eventually adapted in the follow-up period by de novo development) of well-established TCM syndromes and symptoms. Survival was analyzed using Kaplan-Meier and Cox regression. Propensity score analysis (PSA) with matching and inverse probability of treatment weighting (IPTW) were used to adjust for baseline covariate imbalances.

**Results:**

Of 412 patients, 117 received no Moxa, 239 had 1 to 4 treatments, and 56 received >4 treatments alongside conventional treatments. Tumor-Node-Metastasis (TNM) stage IVB and male sex increased dead of disease (DOD)-risk, while TKI-TT, ≥4 Chemotherapy cycles, and Moxa improved survival (*P* < .05). Median survival (MST): Reference group (PBC + CHM) 20.0 months; Moxa 32.0; TKI-TT 33.0; TKI-TT+1–4 Moxa 33.0; TKI-TT+>4 Moxa 40.0 months (all *P* < .05). Cox regression indicated a dosage-dependent Moxa effect (*P* = .0004). Restricted Mean Survival Time (RMST) at 36 months favored >4 Moxa+TKI-TT over TKI-TT (+6.2 months, *P* = .01). PSA confirmed results were not due to baseline covariate imbalance.

**Conclusions:**

Moxibustion may dosage-dependently improve survival in PUAD-IV, both in TKI- and non-TKI-treated patients. Randomized clinical trials (RCT) are needed to confirm this.

## Visual abstract


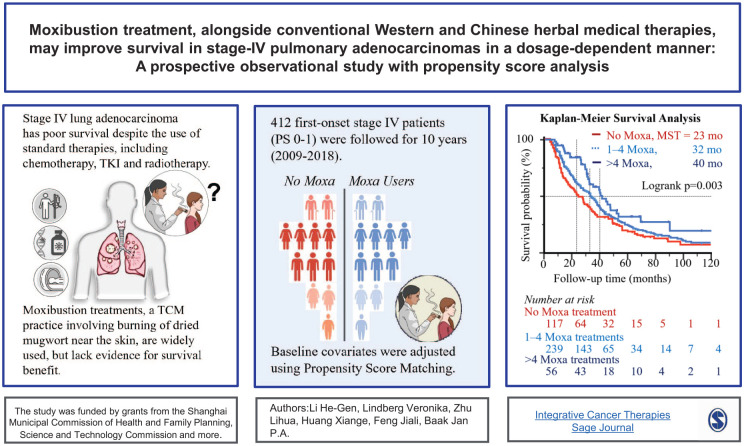


## Introduction

Lung cancer is among the most frequent cancers worldwide and the leading cause of cancer-related mortality, including in China.^1^ The global overall 5-year survival rate of the most common subtype (non-small cell lung cancer = NSCLC) is less than 20%.^2^ Of the 2 million new lung cancers diagnosed worldwide,^3^ 1.7 million are NSCLCs, of which 1 million are in advanced stage IV at the time of diagnosis. Adenocarcinomas are now the most common stage IV histological subtype in many countries, including China, and are biologically and therapeutically different from non-adenocarcinomas. Systemic platinum- based chemotherapy (PBC), targeted therapy (TT), and immunotherapy have greatly improved these patients’ survival rates. However, short-, intermediate-, and long-term survival cannot be accurately predicted with current prognosticators.^4^

In East Asia and elsewhere, many stage IV patients are also treated (in addition to PBC and TT) with oral Chinese herbal medicine (CHM), which can further improve their survival rates.^5^ Moreover, a simple outpatient external traditional Chinese medicine (TCM) therapy called moxibustion^6^ is also often used in cancer treatment for the prevention and treatment of various complications (including cancer-related or therapy-induced symptoms, such as nausea, vomiting, fatigue, and pain) and thereby to enhance quality of life.^7,8^ This approach is increasingly supported by recent research, with Bae et al. (2024) finding that moxibustion alleviates cancer-related fatigue in breast cancer patients (10 RCTs, n = 744),^9^ while Xie et al^10^ demonstrated that combining moxibustion with usual medical care optimally improves quality of life for patients experiencing cancer pain (111 RCTs, n = 9549). Many TCM medical oncologists in China believe that moxibustion treatment (Moxa) is also prognostically useful in cancer, although studies evaluating the survival-improving effect of moxibustion in pulmonary metastatic stage IV adenocarcinomas are lacking. Preclinical studies in animal models, suggest that moxibustion may prolong survival of cancer by overcoming the immunosuppressive microenvironment through inhibiting the PD-1/PD-L1 signaling pathway and inhibit tumor progression.^11^

In a prospective long-term observational analysis of pulmonary stage IV adenocarcinoma patients (all treated with conventional Western and oral TCM treatments), we tested whether Moxa independently improves survival, if so, for how long, and whether the survival-improving effects are Moxa dosage dependent. Moxa was administered to patients by medical oncologists, as indicated at the start of the treatment (and eventually adapted in the follow-up period by de novo development) of well-established TCM syndromes and symptoms.

## Materials and Methods

### Ethics, Patients, Stages, Performance Scores, and Treatments

The ethics, patients, clinical and baseline features, and treatments have been described in previous studies.^4,5^ This is an observational study in which we observed the effect of risk factors, treatments, and other interventions without trying to change who was or was not exposed to it.^12^ The study was approved by the Institutional Research Board of the Longhua University Hospital (LUH), Xuhui District, Shanghai, China (Number 15-LCSY38). The last co-author obtained permission to participate in the study from the research director of the university hospital where he works. The study was performed in accordance with the World Medical Association Declaration of Helsinki^13^ and complied with the principles of the CIOMS International Ethical Guidelines for Biomedical Research Involving Human Subjects.^14^ Before treatment, all patients agreed to participate and signed a consent form for their data to be used in the study, which was kept in their hospital records. There were no changes to their usual treatments at the hospital.

**Figure 1.**
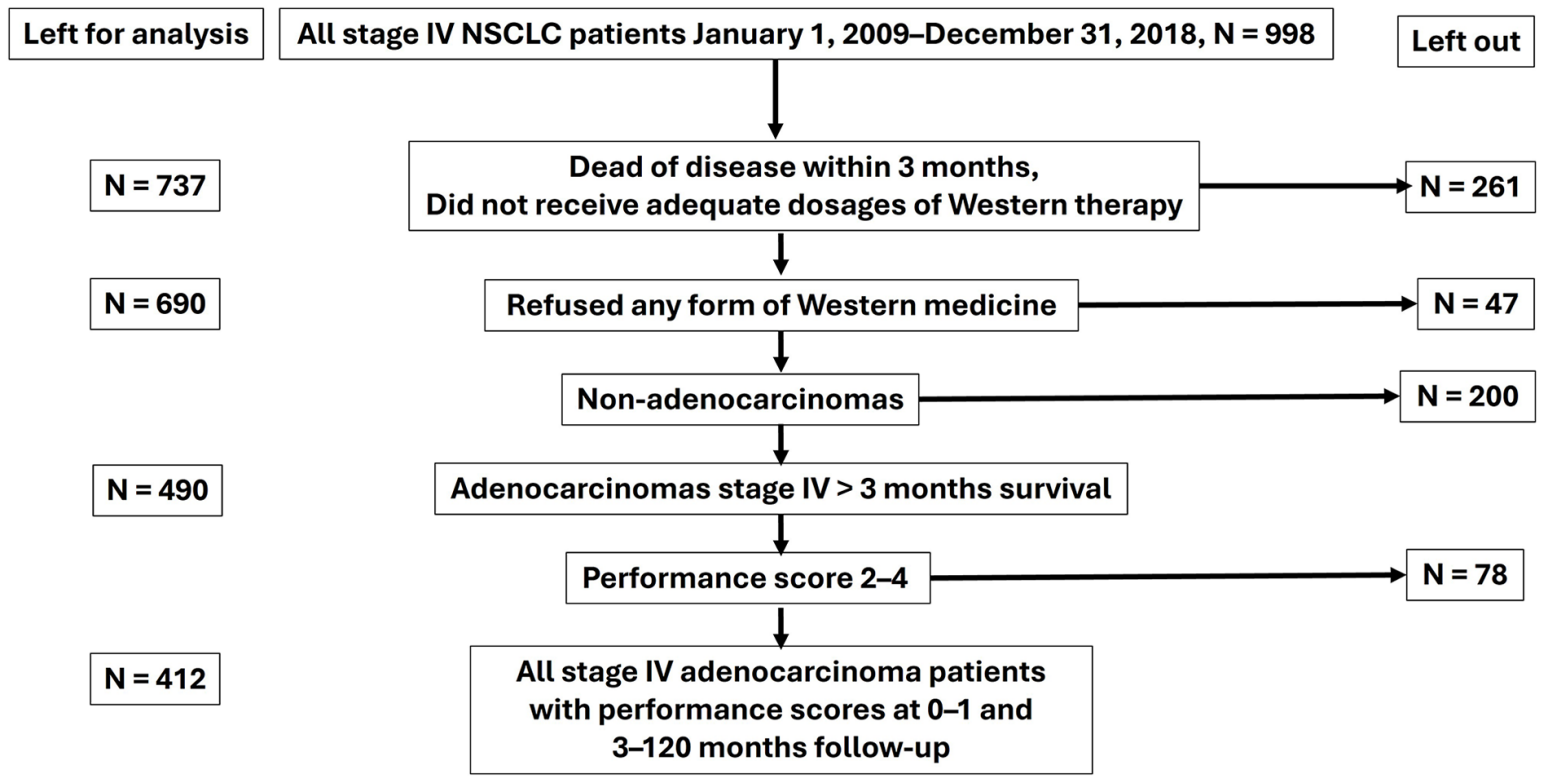
Flowchart of the original, excluded, and included patients in the study.

All originally consecutive 998 pathologically confirmed first-onset stage IV NSCLC patients diagnosed at the LUH between January 1, 2009 and December 31, 2018, were considered. Of these patients, 261 died within 3 months and never got a chance to get adequate Western or TCM treatments, 737 survived 3.2 to 120.0 months, 47 refused any form of PBC and/or TKI-TT and were therefore omitted, which left 690 stage IV NSCLC patients for analysis. As adenocarcinomas and non-adenocarcinoma NSCLCs differ prognostically and therapeutically, all 200 non-adenocarcinomas were omitted. The Eastern Cooperative Oncology Group performance score (PS) was used, defined as 0 to 4.^15^ Adenocarcinoma patients with a performance score of 2 to 4 have a much worse prognosis, often do not receive full conventional Western treatments (in contrast to those with PS 0-1), and sparsely and rarely receive moxibustion treatments. They were also omitted from the study, leaving 412 PS 0-1 adenocarcinoma patients (Figure 1).

The data were retrieved from the patients’ records by 4 experienced medical oncologists, who carefully registered each item. For quality control, in a random 10% of the records, the items were reviewed by another independent medical oncologist. Only minor discrepancies occurred, which were rare, clinically insignificant, and consistently resolved by consensus, supporting the conclusion that the 10% data quality control was satisfactory.

The disease stage was defined according to the eighth edition of the TNM classification for lung cancer as IVA or IVB.^16^ The smoking habit index was the number of cigarettes smoked per day (as indicated by the patient) times the number of years smoked. We used ≤500 versus >500 as the thresholds to distinguish between “no-to-moderate” and “very heavy” smokers.

The patients were randomly treated with 1 of 4 platinum-based regimens (for the details, see Ref.^17^; there were no differences in the outcomes of lung cancer with these different regimens). The standard of care for the initial treatment of advanced NSCLC is 4 to 6 PBC cycles, followed by close observation.^18^ Patients with grade 1 to 3 nausea or vomiting routinely received pharmaceutical anti-emetic ondansetron (Zofran) treatment. In addition, radiotherapy was given for palliative reasons.^19^

The following first-line tyrosine kinase inhibitors for treating advanced lung cancer with epidermal growth factor receptor (EGFR)^18-21^ mutation (+) were used as a primary treatment: gefitinib (Iressa^®^), erlotinib (Tarceva^®^), and icotinib (Conmana^®^). When these TKIs failed, second-line afatinib (Gilotrif^®^) was given, or else a third-generation EGFR-TKI, osimertinib (Tagrisso^®^).

All patients were classified using well-established criteria for TCM syndromes and received oral TCM herbal medicines (250 ml, twice daily) throughout the treatment period, adjusted for any de novo developing TCM syndromes or symptoms. For details of the herbal treatments, see Ref.^20^

### Moxibustion

Moxa was indicated in the follow-up period by the treating medical specialists, based on the patients’ developing TCM syndromes, conditions, and symptoms. The moxibustion techniques have been described in detail.^6^ These methods are traditionally employed to stimulate circulation, enhance the flow of qi (vital energy), and activate meridians, in accordance with the principles of TCM. Moxibustion may also influence physiological pathways similar to those affected by acupuncture, including enhanced circulation, immune modulation, and neural responses.^21-24^ The treatment duration is 15 to 20 minutes. The number of moxibustion treatments were categorically registered for this study as none, 1-4, 4-8, and >8. As the numbers with 4-8 (N = 35) and ≥8 (N = 21) were small, they were grouped together and analyzed as >4 (N = 56).

### Statistical Methods

SPSS version 29 and MedCalc 23.1.7 were used for statistical analyses. Descriptive statistics were assessed for all features. For survival analyses, continuous features were discretized according to medians, tertiles, quartiles, or the results of receiver operating curve (ROC) analysis. DOD and AWD at the last follow-up were the designated endpoints. Single (Kaplan-Meier) and multivariate survival analysis (Cox model: Enter, Forward, Backward, and Stepwise methods) were used, with probabilities of <.05 as the threshold for significance. The hazard ratio (HR) and the 95% confidence interval of the HR (95% CI) were calculated. To evaluate the influence of the duration of follow-up in the survival regression analysis, the medians, tertiles, and quartiles of follow-up durations were used as covariates.

To reduce confounding, predicted probabilities from logistic regression, indicating the likelihood of receiving moxibustion treatment, were used as propensity scores in the PSA.^25-28^ Matching (1:1 greedy algorithm) and IPTW were used to adjust for baseline covariate imbalance^29,30^ (Supplemental Table 1). Covariate balance was assessed using SMDs (<0.1 indicating good balance). Sensitivity analysis^31^ with exact matching (0 vs Any, and 0, 1-4, >4 Moxa groups) ensured consistency and reliability across methods (Supplemental Table 2).

Consistent results across the methods suggest that the findings are stable and not sensitive to variations in matching strictness.

## Results

The median survival time (MST) for the 412 patients was 30.7 months. This may seem high, but it should be kept in mind that these patients came from a total of 998 stage IV NSCLCs, excluding the following groups with a poorer prognosis and different treatments: death <3 months (N = 261), 47 who refused all Western medicines (N = 47), all non-adenocarcinomas (N = 200), and all 78 adenocarcinomas with PS 2-4. The MST for all 998 stage IV NSCLC patients was 12.0 months (consistent with other large studies). Therefore, we conclude that the material is representative.

Supplementary demographics (Supplemental Table 3a) compare baseline characteristics and treatments between No Moxa (N = 117) and Any Moxa (N = 295) for the 412 patients. EGFR mutations were marginally significant (*P* = .07, logistic regression *P* = .047).

Supplementary demographics (Supplemental Table 3b) shows baseline characteristics across Moxa groups (No, 1-4, >4), with EGFR mutations (*P* = .02) and targeted therapy (*P* = .03) being significant.

Propensity scores were calculated using baseline characteristics, achieving excellent balance after PSM (Supplemental Table 4). Standard Mean Differences (SMDs) compared the balancing methods across the full dataset, PSM, IPTW, and stabilized IPTW (Supplemental Table 5, Supplemental Figure 1). PSM improved covariate balance but reduced the sample size (N = 230). IPTW provided a good balance while retaining the full sample size (N = 412). Stabilized IPTW did not meet SMD criteria for TNM stage and EGFR mutation and was not further considered. Sensitivity analysis using 1:1 exact matching for 0 versus Any and 1:1:1 exact matching for the 0, 1-4, and >4 Moxa groups confirmed consistent treatment effects across methods. This indicates that the results are stable and not influenced by variations in matching strictness, thus supporting the reliability of the findings (Supplemental Table 2).

**Figure 2.**
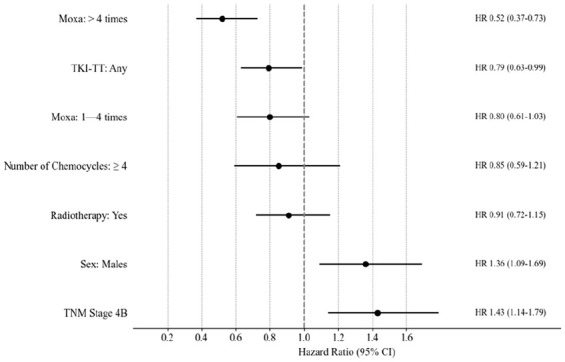
Hazard ratios (HRs) with 95% CI for the most significant baseline features and therapeutic modalities in stage IV adenocarcinoma (N = 412) from Kaplan-Meier survival analysis, using log-rank tests. HR > 1 indicates increased risk, HR<1 suggests reduced risk. Narrower CIs indicate more precise estimates.

To explore the impact of individual factors on survival, univariate analyses were conducted. The results of the characteristics of the 412 patients are shown in Table 1 and Figure 2.

**Table 1.** The Results of Univariate Kaplan-Meier Survival Analysis of the Characteristics, With Log-Rank Values for the Probabilities of No Difference, Hazard Ratios, and 95% Confidence Intervals, Studied in the 412 Stage IV Adenocarcinomas Patients With a Performance Score of 0-1 and 3-120 Months Follow-Up.

Characteristic	Dead of disease/at risk	% Censored (alive with disease)	Median survival time (months)	Probability of no difference	Hazard ratio (95% confidence interval)
*Baseline features*					
Total	323/412	22%	30.7		
Follow-up duration					
Patients alive with disease	0/89		34		
Patients dead of disease	323/323		23		
Age (years)					
28-55 (Ref^a^)	95/113	16%	33.0		
56-62	81/103	21%	30.0	.66	1.07 (0.79-1.44)
63-68	76/104	27%	27.8	.45	1.13 (0.83-1.53)
69-81	71/92	23%	36.0	.96	0.99 (0.73-1.35)
<75 (Ref^a^)	297/380	22%	31.0		
≥75	26/32	19%	23.8	.42	1.20 (0.78-1.85)
Sex					
Females (Ref^a^)	156/207	25%	35.0		
Males	367/205	19%	25.9	.007	1.36 (1.09-1.69)
Smoking Habit Index					
No to mild (Ref^a^)	266/335	21%	31.0		
Heavy	57/77	26%	30.3	.80	1.04 (0.78-1.39)
TNM stage					
4A (Ref^a^)	109/151	28%	39.0		
4B	214/261	18%	24.3	.002	1.43 (1.14-1.79)
TCM syndromes					
Deficiency of lung and spleen (Ref^a^)	166/200	17%	28.0		
Endogenous heat due to yin deficiency	9/9	0%	26.0	.64	1.19 (0.57-2.46)
Deficiency of qi and yin	141/188	25%	31.2	.20	0.86 (0.69-1.08)
Deficiency of spleen and kidney	7/15	53%	71.0	.12	0.63 (0.35-1.13)
EGFR mutations					
None	290/353	18%	28.8		
Any	33/59	44%	40.0	.07	0.74 (0.54-1.02)
*Therapeutic modalities*					
Radiotherapy					
No (Ref^a^)	214/279	23%	30.0		
Yes	109/133	18%	33.0	.42	0.91 (0.72-1.15)
Number of chemo cycles					
<4 (Ref^a^)	39/52	25%	28.0		
4-6	284/360	21%	31.2	.35	0.85 (0.59-1.21)
TKI targeted therapy					
None (Ref^a^)	208/259	20%	27.8		
Tarceva	26/30	13%	25.9	.80	0.95 (0.63-1.42)
Iressa	72/98	27%	38.0	.04	0.77 (0.59-0.99)
Conmana	13/21	38%	55.9	.05	0.64 (0.41-1.01)
Second Line TKI Osimertinib, Afatinib	4/4	0%	8.0	<.0001	223.08 (18.56-2680.52)
TKI Targeted therapy					
None (Ref^a^)	208/259	20%	27.8		
Any	115/153	25%	36.0	.04	0.79 (0.63-0.99)
Moxibustion, no. of treatments					
None (Ref^a^)	104/117	11%	23.0		
1-4 times	186/239	22%	32.1	.056	0.80 (0.61-1.03)
>4 times	33/56	41%	40.0	.003	0.52 (0.37-0.73)
None (Ref^a^)	104/117	11%	23.0		
≥1 times	219/295	26%	33.0	.009	0.72 (0.56-0.92)
1-4 times	186/239	22%	32.1		
>4 times	33/56	41%	40.0	.02	0.68 (0.49-0.94)
0-4 times (Ref^a^)	290/356	19%	28.0		
>4 times	33/56	41%	40.0	.005	0.65 (0.48-0.88)
None (Ref^a^)	104/117	11%	23.0		
>4 times	33/56	41%	40.0	.001	0.56 (0.39-0.79)

Ref^a^: This subgroup is used for this characteristic as the reference group to calculate the significance, hazard ratios, and 95% CI calculations with the other subgroups.

Abbreviation: TKI, tyrosine kinase inhibitors.

TNM Stage 4B and male sex show a higher mortality risk (*P* = .002, .007).

Targeted therapy (None vs Any *P* = .04) and Moxa are associated with improved survival, with more frequent Moxa (>4 times) showing a stronger protective effect (*P* = .001). Palliative radiotherapy showed no prognostic significance (MST: 30 vs 33 months, *P* = .42; Table 1). Multivariate Cox regression identified a significant impact of ≥4 Chemotherapy cycles on survival (HR = 0.63, *P* = .032, Supplemental Table 6a).

**Figure 3.**
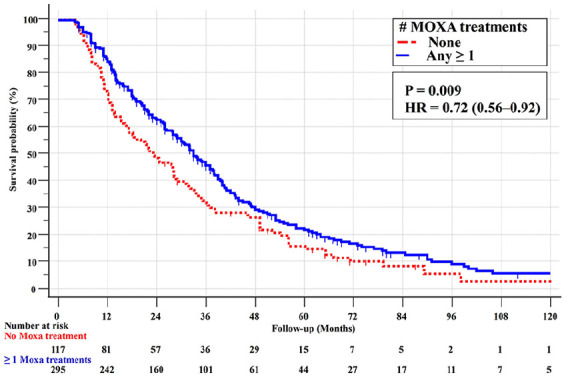
Kaplan-Meier survival curves (with log-rank test) for the 412 stage IV PS = 0-1 adenocarcinoma patients with none and any (≥1) moxibustion treatments.

The Kaplan-Meier survival curve in Figure 3 confirmed a significant survival benefit with moxibustion, with MST of 23 months for no moxibustion and 33 months for any moxibustion (*P* = .009, HR = 0.72, 95% CI 0.56-0.92).

**Figure 4.**
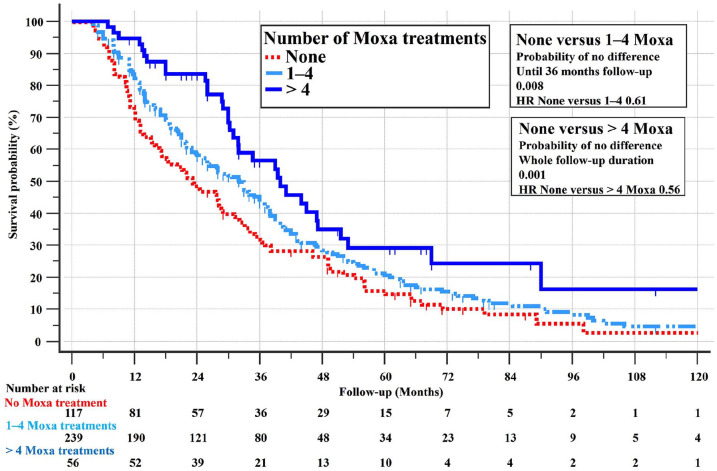
Survival curves for the 412 stage IVPS = 0-1 adenocarcinoma patients with none, 1-4, and >4 moxibustion treatments.

As presented in Figure 4, more frequent moxibustion (1-4 vs >4 treatments) further improved survival, with MSTs of 32.1 versus 40.0 months (*P* = .02, HR = 0.68, 95% CI 0.49-0.94).

Cox proportional hazards regression analyses were performed on the full dataset (N = 412), with IPTW weights (N = 412) and on the propensity score matched dataset (N = 230; Supplemental Tables 6a, 6b, and 6c). All balancing approaches produced consistent results, affirming the robustness of the findings and demonstrating a significant dose- dependent association between Moxa and improved survival both alone and alongside TKI-TT.

To further explore the impact of Moxa dosage on survival, we analyzed the differences among 3 Moxa treatment subgroups, as well as the comparison between any Moxa use versus none (Supplemental Table 7, column 5). Significant survival differences were observed during the 12- to 60-month follow-up, including none versus any, none versus 1-4 treatments, none versus >4 treatments, and 1-4 versus >4 treatments (all *P* < .05).

**Figure 5.**
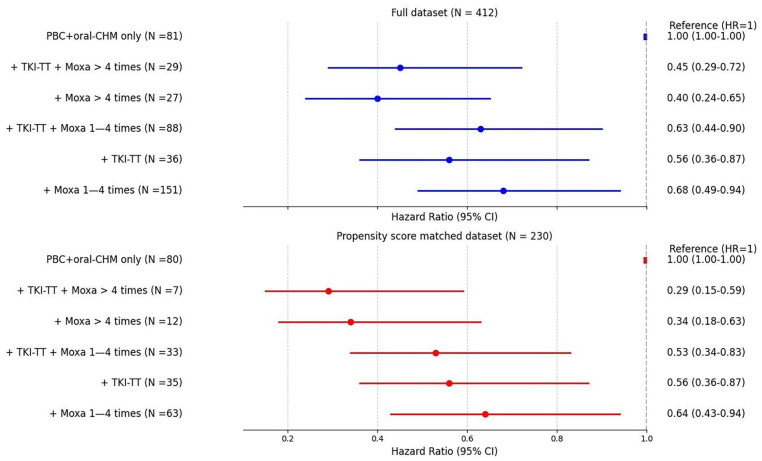
Multivariate survival analysis: Moxa and TKI-TT in stage IV adenocarcinoma. Hazard ratios with 95% CIs for full (N = 412) and propensity score matched (N = 230) datasets. This figure highlights the survival benefits of Moxa and TKI-TT, both individually and in combination, compared to standard care (PBC + oral CHM only).

Additionally, we investigated the combined and individual effects of Moxa and TKI-TT on survival outcomes (Supplemental Tables 8a, 8b). The overall MST was 30.7 months. The reference group (standard care, PBC + CHM, N = 81) had an MST of 20.0 months; adding Moxa alone (N = 175) increased MST to 32.0 months, TKI-TT alone to 33.0 months; combining TKI-TT with 1-4 Moxa also 33.0 months (although in a larger subgroup: 88 vs 36); TKI-TT + >4 Moxa (N = 29) to 40.0 months (all *P* < .05). Combining Moxa>4 times and TKI-TT provided the greatest survival benefit (Figure 5). RMST showed >4 Moxa with TKI-TT outperformed TKI-TT alone between 12 and 48 months (Supplemental Table 9a), with similar findings between 24 and 60 months in the matched dataset (Supplemental Table 9b). At 36 months, RMST favored >4 Moxa + TKI-TT over TKI-TT alone, with +6.18-months survival benefit (*P* = .01).

The start dates for the individual Moxa were not registered, as the selection of patients for Moxa treatments is based on the development of new TCM symptoms in the follow-up, not at a certain point in time. However, the correlation between the number of Moxa treatments and the TCM syndromes at the first Stage IV adenocarcinoma diagnosis was not significant (*P* = .14).

Overall, PSA, with adjustments for confounding and sensitivity analysis, suggests that the results were not influenced by the possibility that longer survival increases the likelihood of receiving treatment.

## Discussion

This observational study suggests that in patients with stage IV pulmonary adenocarcinoma (PS 0-1), Moxa (None vs Any), in addition to PBC and/or TKI-TT and oral CHM, may improve survival. A comparison of 1-4 versus >4 Moxa treatments shows further survival improvement with >4 Moxa, suggesting a dosage-response relationship. Positive aspects of the study include the large sample size, long follow-up, standardized treatments, and ease of administering moxibustion. However, independent RCTs are needed to validate these findings.

Several important questions remain. First, the situation of the patients having to pay extra for Moxa may have caused the selection of economically favored patients.^32^ However, Moxa costs in China are very low (50 RMB), and the Chinese co-authors, who are also medical oncologists, had never experienced patients refusing moxibustion treatments due to cost.

Second, unmeasured variables other than Moxa may have caused improved survival. After the PSA with IPTW balancing, baseline characteristics (including TCM syndromes, EGFR mutations, and targeted therapy) were not correlated with Moxa, which was prescribed later. However, unmeasured baseline features may have had an effect. We are not aware of published predictors in cancer patients regarding Moxa treatment. However, for other diseases, such as first-time strokes—which are increasing among the Taiwanese—it was found that the choice of TCM treatment was associated with clinical and socioeconomic characteristics and high education.^33^ In addition, Malaysian *females* with metabolic syndrome were more likely to use TCM.^34^ Such factors should therefore be included in future RCT studies evaluating the prognostic effect of Moxa in stage IV NSCLC patients.

Third, patients in the current study may have sought alternative treatments without informing their TCM specialists. A recent study highlighted the widespread use of complementary and alternative medicine (CAM) among Norwegians in the 12-month period before they were interviewed, also alongside patients with cancer treatments.^35^ Our patients received oral CHM, PBC and/or targeted therapy, and moxibustion, leaving few other options in Shanghai, as Qi Gong healing therapy was not used in China during the enrollment period due to regulatory restrictions. While we lack data on dietary supplements, based on clinical experience, their use was likely low. In other countries, this may be different. These factors should be explored in future RCTs.

In previous clinical cancer-related studies, Moxa reduced therapy-induced nausea, vomiting, fatigue, and pain and enhanced quality of life.^7^ Because many patients find moxibustion pleasant, it may offer benefits beyond survival improvement and thus deserves more research. Adding >4 Moxa to TKI-TT showed a significant increase in MST from 33 to 40 months (*P* = .01).

Regarding the working mechanisms of moxibustion, at the cellular level, it may enhance immune function by increasing the activity and number of natural killer (NK) cells, lymphocytes, and macrophages.^36,37^ Additionally, moxibustion reduces levels of pro-inflammatory cytokines, such as IL-1, IL-6, and TNF-alpha, potentially mitigating cancer pain.^21,22^ Research on acupuncture (which may share some mechanisms with Moxa) has shown significant increases in oxygenated hemoglobin (oxy-Hb) and total hemoglobin (t-Hb) in stimulated regions, indicating improved blood flow and tissue oxygenation. These effects may also occur with Moxa.^23^ This improved circulation might enhance the efficacy of chemotherapy by facilitating the delivery of therapeutic agents to tumor sites. In a rat model, moxibustion influenced gene expression.^24^ Key findings include the regulation of circRNA_02767, rno-miR-483-3p, and Gfap. In squamous cell carcinoma, miR-483-3p sensitized cells to apoptosis by targeting anti-apoptotic genes such as API5, BIRC5, and RAN. Modulation of circRNA-miRNA-mRNA networks could be a relevant area for future research to explore its impact on cancer-related pathways. More fundamental research is necessary to confirm these effects.

The safety of smoke from moxibustion has been debated, with some reports suggesting potential harm. These potential harms include allergic reactions (skin flushing, swelling, itching, or rashes) potentially due to sensitivity to mugwort, the herb used in moxibustion; burns from improper technique; and rarely, infections at treatment sites.^38,39^ Traditional moxa smoke can cause respiratory symptoms like coughing, headaches, and dry throat, and may contain harmful pollutants like nitrogen oxides and carbon monoxide, especially in poorly ventilated settings. However, studies show that under normal conditions, the volatile matter and carbon monoxide levels remain within safe limits.^40^ Smokeless alternatives, such as infrared moxibustion (3.95 µm), have been developed to address smoke-related concerns; however, skin reactions may still occur with any form of heat-based therapy.^41^

Observational studies play an important role in advancing medical knowledge, as they may provide useful data and spark new areas of investigation by prospective RCT, which is what hopefully will happen with the current data. In a thorough comparison in 2014 of healthcare outcomes assessed with observational study designs compared with those assessed in randomized trials, of the 14 reviews, 11 (79%) found no significant difference between observational studies and RCTs, regardless of the specific observational study design, heterogeneity, or inclusion of studies of pharmacological interventions.^42^ A recent study confirmed these conclusions.^43^ However, it is important to emphasize that independent RCTs are necessary to validate the current results.

## Conclusions

Moxibustion may improve, in a dosage-dependent manner, survival in stage IV lung adenocarcinoma, both in TKI- and non-TKI-treated patients. However, further RCTs are necessary to confirm its effectiveness and rule out confounding factors.

## Supplemental Material

sj-docx-3-ict-10.1177_15347354251342739 – Supplemental material for Moxibustion Treatment, Alongside Conventional Western and Chinese Herbal Medical Therapies, May Improve Survival in Stage-IV Pulmonary Adenocarcinomas in a Dosage-Dependent Manner: A Prospective Observational Study With Propensity Score Analysis Supplemental material, sj-docx-3-ict-10.1177_15347354251342739 for Moxibustion Treatment, Alongside Conventional Western and Chinese Herbal Medical Therapies, May Improve Survival in Stage-IV Pulmonary Adenocarcinomas in a Dosage-Dependent Manner: A Prospective Observational Study With Propensity Score Analysis by Hegen Li, Veronika Lindberg, Lihua Zhu, Xiange Huang, Jiali Feng and Jan P. A. Baak in Integrative Cancer Therapies

sj-jpg-1-ict-10.1177_15347354251342739 – Supplemental material for Moxibustion Treatment, Alongside Conventional Western and Chinese Herbal Medical Therapies, May Improve Survival in Stage-IV Pulmonary Adenocarcinomas in a Dosage-Dependent Manner: A Prospective Observational Study With Propensity Score Analysis Supplemental material, sj-jpg-1-ict-10.1177_15347354251342739 for Moxibustion Treatment, Alongside Conventional Western and Chinese Herbal Medical Therapies, May Improve Survival in Stage-IV Pulmonary Adenocarcinomas in a Dosage-Dependent Manner: A Prospective Observational Study With Propensity Score Analysis by Hegen Li, Veronika Lindberg, Lihua Zhu, Xiange Huang, Jiali Feng and Jan P. A. Baak in Integrative Cancer Therapies

sj-jpg-2-ict-10.1177_15347354251342739 – Supplemental material for Moxibustion Treatment, Alongside Conventional Western and Chinese Herbal Medical Therapies, May Improve Survival in Stage-IV Pulmonary Adenocarcinomas in a Dosage-Dependent Manner: A Prospective Observational Study With Propensity Score Analysis Supplemental material, sj-jpg-2-ict-10.1177_15347354251342739 for Moxibustion Treatment, Alongside Conventional Western and Chinese Herbal Medical Therapies, May Improve Survival in Stage-IV Pulmonary Adenocarcinomas in a Dosage-Dependent Manner: A Prospective Observational Study With Propensity Score Analysis by Hegen Li, Veronika Lindberg, Lihua Zhu, Xiange Huang, Jiali Feng and Jan P. A. Baak in Integrative Cancer Therapies
