# Supplementary material for: Moxibustion Treatment, Alongside Conventional Western and Chinese Herbal Medical Therapies, May Improve Survival in Stage-IV Pulmonary Adenocarcinomas in a Dosage-Dependent Manner: A Prospective Observational Study With Propensity Score Analysis
Source: Integr Cancer Ther. 2025 Jun 19;24:15347354251342739. doi: 10.1177/15347354251342739 (PMC12179449; doi:10.1177/15347354251342739)
Supplement: sj-docx-3-ict-10.1177_15347354251342739 – Supplemental material for Moxibustion Treatment, Alongside Conventional Western and Chinese Herbal Medical Therapies, May Improve Survival in Stage-IV Pulmonary Adenocarcinomas in a Dosage-Dependent Manner: A Prospective Observational Study With Propensity S [file sj-docx-3-ict-10.1177_15347354251342739.docx]

**Supplementary Table 1.** Standardized mean difference (SMD) calculation methods by covariate type.

| **Type of Covariate** | **Formula** | **Description** | **Explanation** |
| --- | --- | --- | --- |
| Continuous Covariates | SMD = (Mean₁ - Mean₀) / √[(Var₁ + Var₀) / 2] | Mean₁, Mean₀: Means in treated and control groups  Var₁, Var₀: Variances in treated and control groups | Difference in group Means divided by pooled standard deviation (equivalent to Cohen's d).^1^ |
| Categorical Covariates (2 categories) | SMD = (P₁ - P₀) / √[P(1-P)]  where P = (n₁P₁ + n₀P₀) / (n₁ + n₀) | P₁, P₀: Proportions in treated and control groups  n₁, n₀: Sample sizes in treated and control groups | Difference in proportions divided by SD of pooled proportion. |
| Categorical Covariates (> 2 categories) | SMD_overall = √[Σ(P₁ᵢ - P₀ᵢ)² / K] | P₁ᵢ, P₀ᵢ: Proportions of category i in treated and control groups  K: Number of categories | Root mean squared difference in proportions across categories, giving the average distance between groups. |
| Weighted SMD (IPTW and Stabilized IPTW) | Weighted_Mean = Σ(wᵢ * xᵢ) / Σwᵢ | wᵢ: Weight for observation i  xᵢ: Value of covariate for observation i | In weighted analyses, Means are calculated using propensity score weights instead of equal treatment of all observations. |
| Weighted SMD for Categorical Covariates (2 categories) | Weighted_SMD = (P₁w - P₀w) / √[Pw(1-Pw)]  where Pw = (n₁P₁w + n₀P₀w) / (n₁ + n₀) | P₁w, P₀w: Weighted proportions in treated and control groups | Uses weighted proportions to adjust for sample differences. |
| Weighted SMD for Categorical Covariates (> 2 categories) | Weighted_SMD_overall = √[Σ(P₁wᵢ - P₀wᵢ)² / K] | P₁wᵢ, P₀wᵢ: Weighted proportions of category i in treated and control groups | Calculates the root mean squared difference between weighted proportions across all categories. |

1. The standardized mean difference (SMD) measures group differences in standard deviation units and is widely used to assess covariate balance in observational studies, particularly with propensity score methods. An SMD below 0.1 indicates good balance between groups. Unlike P-values, SMD is independent of sample size. In inverse probability of treatment weighting (IPTW), SMD assesses balance after weighting to help reduce bias.

**Supplementary Table 2.** Quality checklist of propensity score analysis.

| **Item** | **Recommendation** | **Relevant Details** | **Reported in Section** | **Page** |
| --- | --- | --- | --- | --- |
|  | **Preparation for PS Analysis** |  |  |  |
| 1. | Point out the scientific background | Provide context and rationale | Introduction | 3 |
| 2. | Indicate key components of study design | Describe study design, including treatment and control groups, and use of PS to reduce bias | Statistical methods | 4 |
| 3. | State the study objectives | Define specific aims or hypotheses | Introduction | 3 |
| 4. | Describe data sources and variables | Detail data collection and variable definitions | Materials and methods | 3–5 |
|  | **PS Model Building** |  |  |  |
| 5. | Select variables for PS model | Justify selection of confounders | Statistical methods Supplementary Tables 3a, 3b | 5 |
| 6. | Decide PS estimation method | Justify choice of method (e.g., logistic regression) | Statistical methods | 5 |
| 7. | Evaluate overlap | Assess overlap using statistical tests: 10 strata used, no non-overlapping strata found | - | - |
| 8. | Present initial balance diagnostics | Report standardized mean differences and other diagnostics | Statistical methods,  Supplementary Tables 4, 5 Supplementary Figure 1 | 5 |
|  | **Application of PS Methods** |  |  |  |
| 9. | Specify treatment effect type | The treatment effect estimated was the average treatment effect (ATE) for the full dataset and IPTW dataset, and the average treatment effect on the treated (ATT) for the matched dataset. In our large dataset, ATT effectively estimated ATE, as the larger sample size reduced bias. | - | - |
| 10. | State PS method used | Describe PS method (e.g., matching, weighting) | Results | 5 |
| 10a) | Detail matching/weighting strategy | Specify matching ratio or weighting approach | Results | 5 |
| 10b) | Explain management of extreme weights | Describe any techniques used (e.g., trimming): No extreme weights were found. | - | - |
| 10c) | Provide matching details | 1:1 greedy matching SMD < 0.1, p > 0.5. Of 412 patients in the full dataset, 230 were kept in the matched dataset, and 182 were excluded. | Results | 5 |
| 10d) | Describe stratification approach | A stratification approach with 10 strata was used to assess overlap between treatment groups. Stratification was not applied for matching purposes. | - | - |
| 11. | Present subsequent balance diagnostics | Report balance using SMD or other methods post-matching | Results Supplementary Tables 4, 5 Supplementary Figure 1 | 5 |
| 12. | State model for treatment effect | Describe the statistical model used: Cox regression, Kaplan Meier survival analysis | Result | 5–9 |
| 13. | Perform sensitivity analysis^1^ | To test robustness, a sensitivity analysis with 1:1 exact matching for 0 vs. Any, and 1:1:1 exact matching for the 0, 14, and > 4 Moxa groups (N = 75; 25 per group) confirmed the treatment effects observed in the full dataset. This indicates that the results are stable and not influenced by variations in matching strictness, supporting the reliability of the findings | Results | 5 |
| 14. | Report and interpret treatment effects | Present and interpret effects for all datasets | Result  Table 1  Figures 1–5  Supplementary Figure 2  Supplementary Tables 6a, 6b, 6c, 7, 8a, 8b, 9a, 9b Discussion | 5–9 |

1. Sensitivity analysis in propensity score analysis (PSA) is a crucial step because it helps researchers explore to what extent the estimated treatment effects are robust to potential unmeasured confounding variables or hidden biases, and to enhance the validity of the conclusions regarding treatment effects. In other words, it examines how sensitive the results are to violations of the assumption that all relevant confounders have been controlled for.

**Supplementary Table 3a.** Demographics of baseline and therapy characteristics in stage IV adenocarcinoma patients with performance score 0–1 (N = 412), comparing “None” versus “Any” Moxa.

| **Characteristic/ Category** | **No Moxa (N, %)** | **Any Moxa (N, %)** | **Total (N)** |  | **P-value^1^**  **(Chi-square, None vs. Any Moxa)** | **P-value^2^  (Logistic regression,  None vs.  Any Moxa)** |
| --- | --- | --- | --- | --- | --- | --- |
| **Total (N)** | 117 | 239 | 412 |  |  |  |
| **Age (Years)** |  |  |  |  |  |  |
| 28–55 | 28 (23%) | 85 (29%) | 113 |  |  |  |
| 56–62 | 29 (25%) | 74 (25%) | 103 |  |  | .50 |
| 63–68 | 29 (25%) | 75 (25%) | 104 |  |  | .54 |
| 69–81 | 31 (27%) | 61 (21%) | 92 |  | .57 | .26 |
| **Sex** |  |  |  |  |  |  |
| Female | 61 (52%) | 146 (49%) | 207 |  |  |  |
| Male | 56 (48%) | 149 (51%) | 205 |  | .63 | .60 |
| **TNM Stage** |  |  |  |  |  |  |
| Stage IVA | 36 (31%) | 115 (39%) | 151 |  |  |  |
| Stage IVB | 81 (69%) | 180 (61%) | 261 |  | .12 | .10 |
| **Smoking Habit Index** |  |  |  |  |  |  |
| No to mild | 98 (84%) | 237 (80%) | 335 |  |  |  |
| Heavy | 19 (16%) | 58 (20%) | 77 |  | .42 | .42 |
| **EGFR Mutations** |  |  |  |  |  |  |
| Absent | 106 (91%) | 247 (84%) | 353 |  |  |  |
| Present | 11 (9%) | 48 (16%) | 59 |  | .07 | .047^3^ |
| **TCM Syndromes** |  |  |  |  |  |  |
| Deficiency of lung and spleen | 55 (47%) | 145 (49%) | 200 |  |  |  |
| Endogenous heat due to yin deficiency | 0 (0%) | 9 (3%) | 9 |  |  | .99 |
| Deficiency of qi and yin | 55 (47%) | 133 (45%) | 188 |  |  | .53 |
| Deficiency of spleen and kidney | 7 (6%) | 8 (3%) | 15 |  | .10 | .16 |
| **Radiotherapy** |  |  |  |  |  |  |
| No | 80 (68%) | 199 (68%) | 279 |  |  |  |
| Yes | 37 (32%) | 96 (32%) | 133 |  | .86 | - |
| **Chemotherapy Cycles** |  |  |  |  |  |  |
| < 4 | 14 (12%) | 38 (13%) | 52 |  |  |  |
| 4–6 | 103 (88%) | 257 (87%) | 360 |  | .80 | - |
| **TKI Targeted therapy** |  |  |  |  |  |  |
| None | 81 (69%) | 178 (60%) | 259 |  |  |  |
| Tarceva | 8 (7%) | 22 (8%) | 30 |  |  |  |
| Iressa | 19 (16%) | 79 (27%) | 98 |  |  |  |
| Conmana | 8 (7%) | 13 (4%) | 21 |  |  |  |
| Second Line TKI Osimertinib, Afatinib | 1 (1%) | 3 (1%) | 4 |  | .20 | - |
| **TKI Targeted Therapy** |  |  |  |  |  |  |
| None | 81 (69%) | 178 (60%) | 259 |  |  |  |
| Any | 36 (31%) | 117 (40%) | 153 |  | .10 | - |

*P-values* represent the probability of no significant difference between the “None” and “Any” Moxa.

1. These P-values are derived from univariate chi-square tests, with the first category serving as the reference. This assesses the direct relationship between each variable and Moxa. For categorical variables with multiple levels, the P-value represents an overall comparison.
2. These P-values are based on multivariate logistic regression analysis, assessing the likelihood of receiving Moxa. This analysis accounts for multiple variables simultaneously, potentially revealing relationships masked in univariate analysis. Propensity scores generated from the multivariate logistic regression were used for balancing the groups in subsequent analyses, aiming to reduce confounding effects and improve the validity of comparative survival analyses. Treatment variables were not included in this model.
3. EGFR mutations reached statistical significance (P = 0.047) in logistic regression analysis, compared to the univariate result (P = 0.07), suggesting a higher likelihood of EGFR mutations in patients receiving Moxa. However, the contingency coefficient (0.088) from the Chi-squared test indicates a very weak association. No other baseline characteristics showed statistically significant differences between groups.

**Supplementary Table 3b.** Demographics of baseline characteristics and therapies in stage IV adenocarcinoma patients with performance score 0–1 (N = 412), comparing “None” “1–4,” and “> 4” Moxa.

| **Characteristic/ Category** | **No Moxa (N, %)** | **1–4 times Moxa (N, %)** | **> 4 times Moxa (N, %)** | **Total (N)** | **P-value^1^  0 vs.**  **1–4 vs. > 4**  **Moxa** | **P-value^2^  0 vs.  1–4**  **Moxa** | **P-value^2^ 0 vs.**  **> 4**  **Moxa** | **P-value^2^  1–4 vs. > 4**  **Moxa** |
| --- | --- | --- | --- | --- | --- | --- | --- | --- |
| **Total (N)** | 117 | 239 | 56 | 412 |  |  |  |  |
| **Age (Years)** |  |  |  |  |  |  |  |  |
| 28–55 | 28 (24%) | 69 (28%) | 16 (29%) | 113 |  |  |  |  |
| 56–62 | 29 (25%) | 61 (26%) | 13 (23%) | 103 |  |  |  |  |
| 63–68 | 29 (25%) | 61 (26%) | 14 (25%) | 104 |  |  |  |  |
| 69–81 | 31 (26%) | 48 (20%) | 13 (23%) | 92 | .89 | .53 | .91 | .96 |
| **Sex** |  |  |  |  |  |  |  |  |
| Female | 61 (52%) | 120 (50%) | 26 (46%) | 207 |  |  |  |  |
| Male | 56 (48%) | 119 (50%) | 30 (54%) | 205 | .78 | .73 | .48 | .61 |
| **TNM Stage** |  |  |  |  |  |  |  |  |
| Stage IVA | 36 (31%) | 92 (39%) | 23 41%) | 151 |  |  |  |  |
| Stage IVB | 81 (69%) | 147 (61%) | 33 (59%) | 261 | .28 | .15 | .18 | .72 |
| **Smoking Habit Index** |  |  |  |  |  |  |  |  |
| No to mild | 98 (84%) | 196 (82%) | 41 (73%) | 335 |  |  |  |  |
| Heavy | 19 (16%) | 43 (18%) | 15 (27%) | 77 | .29 | .68 | .10 | .14 |
| **EGFR Mutations** |  |  |  |  |  |  |  |  |
| Absent | 106 (91%) | 205 (86%) | 42 (75%) | 353 |  |  |  |  |
| Present | 11 (9%) | 34 (14%) | 14 (25%) | 59 | .02^3^ | .20 | .01^3^ | .05^3^ |
| **TCM Syndromes** |  |  |  |  |  |  |  |  |
| Deficiency of lung and spleen | 55 (47%) | 121 (51%) | 24 (43%) | 200 |  |  |  |  |
| Endogenous heat due to yin deficiency | 0 (0%) | 9 (4%) | 0 (0%) | 9 |  |  |  |  |
| Deficiency of qi and yin | 55 (47%) | 101 (42%) | 32 (57%) | 188 |  |  |  |  |
| Deficiency of spleen and kidney | 7 (6%) | 8 (3%) | 0 (0%) | 15 | .10 | .10 | .12 | .08 |
| **Radiotherapy** |  |  |  |  |  |  |  |  |
| No | 80 (68%) | 157 (66%) | 42 (75%) | 279 |  |  |  |  |
| Yes | 37 (32%) | 82 (34%) | 14 (25%) | 133 | .40 | .61 | .37 | .18 |
| **Chemotherapy Cycles** |  |  |  |  |  |  |  |  |
| < 4 | 14 (12%) | 30 (13%) | 8 (14%) | 52 |  |  |  |  |
| 4–6 | 103 (88%) | 209 (87%) | 48 (86%) | 360 | .91 | .88 | .67 | .73 |
| **TKI Targeted therapy** |  |  |  |  |  |  |  |  |
| None | 81 (69%) | 151 (63%) | 27 (48%) | 259 |  |  |  |  |
| Tarceva | 8 (7%) | 17 (7%) | 5 (9%) | 30 |  |  |  |  |
| Iressa | 19 (16%) | 58 (23%) | 21 (37%) | 98 |  |  |  |  |
| Conmana | 8 (7%) | 11 (5%) | 2 (4%) | 21 |  |  |  |  |
| Second Line TKI Osimertinib, Afatinib | 1 (1%) | 2 (2%) | 1 (2%) | 4 | .17 | .47 | .02^4^ | .25 |
| **TKI Targeted Therapy** |  |  |  |  |  |  |  |  |
| None | 81 (69%) | 151 (63%) | 27 (48%) | 259 |  |  |  |  |
| Any | 36 (31%) | 88 (37%) | 29 (52%) | 153 | .03^4^ | .26 | .01^4^ | .04^4^ |

*P-values* represent the probability of no significant difference between Moxa treatment groups (“None,” “1–4 times,” and “> 4 times”).

1. Overall comparison: P-values are derived from univariate chi-square tests comparing all three Moxa groups simultaneously for each characteristic. For categorical variables with multiple levels, a single P-value is calculated for the overall comparison. The first category listed for each characteristic is used as the reference.
2. Pairwise comparison: P-values are derived from univariate chi-square pairwise comparisons between the groups (“None” vs. “1–4 times,” “None” vs. “> 4 times,” and “1–4 times” vs. “> 4 times”). The first category listed for each characteristic is used as the reference.
3. EGFR mutations were significantly associated with MOXA treatment (P = 0.02, contingency coefficient = 0.068). In the “> 4 times” group, EGFR mutations were more frequent compared to both the “None” group (P = 0.01, contingency coefficient = 0.2, weak association) and the “1–4 times” group (P = 0.0497, contingency coefficient = 0.1, very weak association).
4. TKI-targeted therapies showed a significant association with MOXA treatment (P range: 0.01 to 0.04), with weak to modest strengths of association as indicated by contingency coefficients ranging from 0.068 to 0.333.

**Supplementary Table 4**: Demographics of baseline characteristics and therapies in stage IV adenocarcinoma patients with performance score 0–1 in propensity score matched dataset (N = 230), comparing “None” “1–4,” and “> 4” Moxa, demonstrating excellent balance between treatment groups following propensity score matching.

| **Characteristic/ Category** | **No Moxa (N, %)** | **1–4 times Moxa (N, %)** | **> 4 times Moxa (N, %)** | **Total (N)** | **P-value^1^  0 vs.**  **1–4 vs. > 4**  **Moxa** | **P-value^2^  0 vs.  1–4**  **Moxa** | **P-value^2^ 0 vs.**  **> 4**  **Moxa** | **P-value^2^  1–4 vs. > 4**  **Moxa** |
| --- | --- | --- | --- | --- | --- | --- | --- | --- |
| **Total (N)** | 115 | 96 | 19 | 230 |  |  |  |  |
| **Age (Years)** |  |  |  |  |  |  |  |  |
| 28–55 | 28 (25%) | 20 (21%) | 3 (16%) | 51 |  |  |  |  |
| 56–62 | 29 (25%) | 20 (21%) | 5 (26%) | 54 |  |  |  |  |
| 63–68 | 29 (25%) | 30 (31%) | 4 (21%) | 63 |  |  |  |  |
| 69–81 | 29 (25%) | 26 (27%) | 7 (37%) | 62 | .80 | .69 | .69 | .68 |
| **Sex** |  |  |  |  |  |  |  |  |
| Female | 59 (51%) | 55 (57%) | 9 (47%) | 123 |  |  |  |  |
| Male | 56 (49%) | 41 (43%) | 10 (53%) | 107 | .59 | .39 | .75 | .43 |
| **TNM Stage** |  |  |  |  |  |  |  |  |
| Stage IVA | 36 (31%) | 28 (29%) | 6 32%) | 70 |  |  |  |  |
| Stage IVB | 79 (69%) | 68 (71%) | 13 68%) | 160 | .94 | .74 | .98 | .83 |
| **Smoking Habit Index** |  |  |  |  |  |  |  |  |
| No to mild | 96 (84%) | 75 (78%) | 17 (89%) | 188 |  |  |  |  |
| Heavy | 19 (16%) | 21 (22%) | 2 (11%) | 42 | .40 | .32 | .51 | .26 |
| **EGFR Mutations** |  |  |  |  |  |  |  |  |
| Absent | 104 (90%) | 85 (89%) | 16 (84%) | 205 |  |  |  |  |
| Present | 11 (10%) | 11 (11%) | 3 (16%) | 25 | .70 | .66 | .41 | .60 |
| **TCM Syndromes** |  |  |  |  |  |  |  |  |
| Deficiency of lung and spleen | 55 (48%) | 46 (48%) | 9 (47%) | 110 |  |  |  |  |
| Endogenous heat due to yin deficiency | 0 (0%) | 0 (0%) | 0 (0%) | 0 |  |  |  |  |
| Deficiency of qi and yin | 55 (48%) | 45 (47%) | 10 (53%) | 110 |  |  |  |  |
| Deficiency of spleen and kidney | 5 (4%) | 5 (5%) | 0 (0%) | 10 | .89 | .96 | .64 | .58 |
| **Radiotherapy** |  |  |  |  |  |  |  |  |
| No | 79 (68%) | 61 (64%) | 14 74%) | 154 |  |  |  |  |
| Yes | 36 (32%) | 35 (36%) | 5 (26%) | 76 | .59 | .43 | .66 | .40 |
| **Chemotherapy Cycles** |  |  |  |  |  |  |  |  |
| < 4 | 14 (12%) | 11 (12%) | 1 (5%) | 26 |  |  |  |  |
| 4–6 | 101 (88%) | 85 (88%) | 18 (95%) | 204 | .68 | .87 | .38 | .42 |
| **TKI Targeted therapy** |  |  |  |  |  |  |  |  |
| None | 80 (69%) | 63 (66%) | 12 (63%) | 155 |  |  |  |  |
| Tarceva | 8 (7%) | 7 (7%) | 1 (5%) | 16 |  |  |  |  |
| Iressa | 18 (16%) | 20 (21%) | 6 (32%) | 44 |  |  |  |  |
| Conmana | 8 (7%) | 4 (4%) | 0 (0%) | 12 |  |  |  |  |
| Second Line TKI Osimertinib, Afatinib | 1 (1%) | 2 (2%) | 0 (0%) | 3 | .71 | .71 | .41 | .72 |
| **TKI Targeted Therapy** |  |  |  |  |  |  |  |  |
| None | 80 (70%) | 63 (66%) | 12 (63%) | 155 |  |  |  |  |
| Any | 35 (30%) | 33 (34%) | 7 (37%) | 75 | .76 | .54 | .58 | .84 |

*P-values* represent the probability of no significant difference between Moxa treatment groups (“None,” “1–4 times,” and “> 4 times”).

1. Overall comparison: P-values are derived from univariate chi-square tests comparing all three Moxa groups simultaneously for each characteristic. For categorical variables with multiple levels, a single P-value is calculated for the overall comparison. The first category listed for each characteristic is used as the reference.
2. Pairwise comparison: P-values are derived from univariate chi-square pairwise comparisons between the groups (“None” vs. “1–4 times,” “None” vs. “> 4 times,” and “1–4 times” vs. “> 4 times”). The first category listed for each characteristic is used as the reference. No statistically significant differences in baseline characteristics and treatments were observed between the groups, indicating excellent balance between treatment groups following propensity score matching.

**Supplementary Table 5.** Evaluation of the effectiveness of different balancing methods in reducing covariate imbalances between treatment groups using standardized mean differences (SMD). SMD values closer to zero indicate better balance, with -0.1< SMD < 0.1 generally considered good balance.

| **Baseline Covariate^1^** | **Full Dataset** | **Propensity Score Matched** | **IPTW Weighted** | **Stabilized IPTW Weighted** |
| --- | --- | --- | --- | --- |
| **Total (N)** | (412) | (230) | (412) | (412) |
| **Age quartile** | -0.11 | 0.10 | 0.04 | 0.09 |
| **Gender** | 0.05 | -0.09 | 0.01 | 0.07 |
| **TNM stage** | -0.17 | 0.04 | 0.02 | 0.19 |
| **Smoking status** | 0.09 | 0.09 | 0.05 | 0.08 |
| **EGFR mutation** | 0.21 | 0.08 | 0.05 | 0.22 |
| **TCM syndrome** | -0.04 | 0.00 | 0.07 | 0.13 |
| **Propensity scores** | 0.45 | 0.01 | 0.04 | 0.35 |

1. This analysis compares the effectiveness of different balancing methods by evaluating SMDs across covariates. The propensity score matching (N = 230) improved covariate balance but reduced the sample size. Inverse probability of treatment weighting (IPTW) achieved good balance while retaining the full sample (N = 412), thus preserving statistical power. Stabilized IPTW also maintained the full sample size but did not sufficiently balance key covariates, particularly TNM stage and EGFR mutation, and was therefore excluded from further analysis. The SMD calculation methodology is detailed in Supplementary Table 1.

**Supplementary Figure 1.** Love plot of standardized mean differences (SMD) across balancing methods.^1^ The IPTW and matched datasets gave optimal SMD results (between -0.1 and +0.1). The IPTW dataset retained all 412 patients, while the matched dataset retained 230 patients.


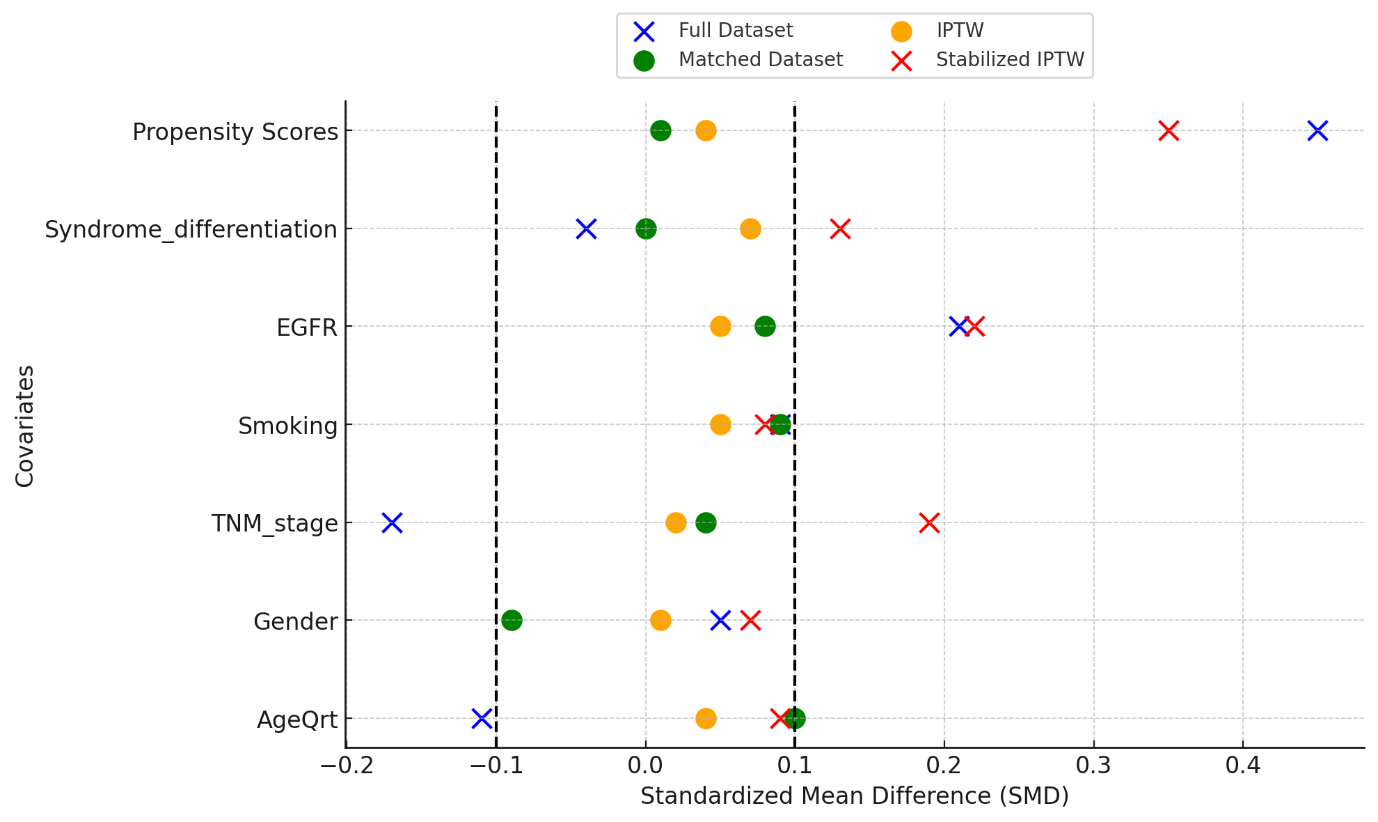


1. Standardized mean differences (SMDs) for various baseline covariates across different balancing methods. SMDs closer to zero indicate better balance between treatment groups, with SMD < 0.1 generally considered good balance. IPTW (inverse probability of treatment weighting) achieved good balance (SMDs < 0.1) for covariates while retaining the full sample size (N = 412), preserving statistical power and generalizability. In contrast, propensity score matching improved balance but at the cost of reducing the sample size. Stabilized IPTW failed to meet the SMD < 0.1 criterion for several covariates, including TNM stage and EGFR mutation, and was excluded from further analysis due to inadequate balance.

**Supplementary Table 6a.** Impact of baseline characteristics and treatments on survival: Cox proportional-hazards regression analysis in stage IV adenocarcinoma patients (N = 412).

| **Characteristic/Category** | **Total  (N = 412)** | **P-value^1^** | **Hazard ratio** | **95% CI  of Hazard ratio** |
| --- | --- | --- | --- | --- |
| **Age (Years)** |  |  |  |  |
| 28–55 | 113 | - | 1.00 | Reference |
| 56–62 | 103 | .28 | 1.19 | 0.87–1.62 |
| 63–68 | 104 | .24 | 1.21 | 0.88–1.65 |
| 69–81 | 92 | .88 | 0.97 | 0.71–1.34 |
| **Sex** |  |  |  |  |
| Female | 207 | - | 1.00 | Reference |
| Male | 205 | .004 | 1.44 | 1.12–1.84 |
| **TNM Stage** |  |  |  |  |
| Stage IVA | 151 | - | 1.00 | Reference |
| Stage IVB | 261 | .002 | 1.49 | 1.16–1.91 |
| **Smoking Habit Index** |  |  |  |  |
| No to mild | 335 | - | 1.00 | Reference |
| Heavy | 77 | .36 | 0.86 | 0.63–1.18 |
| **EGFR Mutations^2^** |  |  |  |  |
| Absent | 353 | - | 1.00 | Reference |
| Present | 59 | .37 | 0.83 | 0.55–1.26 |
| **TCM Syndromes** |  |  |  |  |
| Deficiency of lung and spleen | 200 | - | 1.00 | Reference |
| Endogenous heat due to yin deficiency | 9 | .45 | 1.31 | 0.65–2.62 |
| Deficiency of qi and yin | 188 | .15 | 0.84 | 0.66–1.07 |
| Deficiency of spleen and kidney | 15^3^ | .028 | 0.42 | 0.19–0.91 |
| **Radiotherapy** |  |  |  |  |
| No | 279 | - | 1.00 | Reference |
| Yes | 133 | .19 | 0.85 | 0.66–1.08 |
| **Chemotherapy Cycles^4^** |  |  |  |  |
| < 4 | 52 | - | 1.00 | Reference |
| 4–6 | 360 | .032 | 0.63 | 0.41–0.96 |
| **TKI Targeted therapy** |  |  |  |  |
| None | 259 | - | 1.00 | Reference |
| Tarceva | 30 | .86 | 0.96 | 0.61–1.50 |
| Iressa | 98 | .18 | 0.81 | 0.60–1.10 |
| Conmana | 21 | .016 | 0.47 | 0.26–0.87 |
| Second Line TKI Osimertinib, Afatinib | 4^3^ | .0003 | 7.03 | 2.47–20.00 |
| **Moxa^5^** |  |  |  |  |
| None | 117 | - | 1.00 | Reference |
| 1–4 times | 239 | .014 | 0.73 | 0.57–0.94 |
| > 4 times | 56 | .0004 | 0.49 | 0.33–0.73 |

1. Multivariate Cox regression analyzed baseline characteristics and treatment effects on survival. P-values indicate the probability of no significant difference, with the first category for each characteristic serving as the reference. Hazard ratios and 95% confidence intervals (CI) show the relative risk of death compared to the reference. Key findings include significant impacts on survival according to sex, TNM stage, chemotherapy cycles, certain TKI therapies, and Moxa frequency. This multivariate analysis adjusts for multiple variables, yielding slightly different P-values and HRs compared to the univariate analyses presented in Table 1.
2. EGFR mutations, significant in baseline characteristics, were not predictive of survival in this multivariate model.
3. Results for categories with small patient numbers should be interpreted cautiously.
4. Multivariate Cox regression confirmed the findings from Table 1 and additionally identified a significant impact of the number of chemotherapy cycles on survival.
5. Moxa demonstrated a dose-dependent protective effect, with increased frequency associated with improved survival outcomes in both univariate and multivariate analyses.

**Supplementary Table 6b.** Impact of baseline characteristics and treatments on survival: Cox proportional-hazards regression analysis with IPTW as weights^1^ in stage IV adenocarcinoma patients (N = 412).

| **Characteristic/  Category** | **Total  (N = 412)** | **P-value^2^** | **Hazard ratio** | **95% CI  of Hazard ratio** |
| --- | --- | --- | --- | --- |
| **Age (Years)** |  |  |  |  |
| 28–55 | 113 | - | 1.00 | Reference |
| 56–62 | 103 | .84 | 1.03 | 0.75–1.43 |
| 63–68 | 104 | .19 | 1.23 | 0.90–1.67 |
| 69–81 | 92 | .70 | 0.94 | 0.69–1.29 |
| **Sex** |  |  |  |  |
| Female | 207 | - | 1.00 | Reference |
| Male | 205 | .01 | 1.41 | 1.10–1.81 |
| **TNM Stage** |  |  |  |  |
| Stage IVA | 151 | - | 1.00 | Reference |
| Stage IVB | 261 | <.005 | 1.48 | 1.15–1.91 |
| **Smoking Habit Index** |  |  |  |  |
| No to mild | 335 | - | 1.00 | Reference |
| Heavy | 77 | .49 | 0.89 | 0.64–1.24 |
| **EGFR Mutations** |  |  |  |  |
| Absent | 353 | - | 1.00 | Reference |
| Present | 59 | .26 | 0.77 | 0.49–1.22 |
| **TCM Syndromes** |  |  |  |  |
| Deficiency of lung and spleen | 200 | - | 1.00 | Reference |
| Endogenous heat due to yin deficiency | 9 | .50 | 1.19 | 0.72–1.98 |
| Deficiency of qi and yin | 188 | .18 | 0.84 | 0.65–1.08 |
| Deficiency of spleen and kidney | 15^3^ | .01 | 0.46 | 0.27–0.80 |
| **Radiotherapy** |  |  |  |  |
| No | 279 | - | 1.00 | Reference |
| Yes | 133 | .12 | 0.81 | 0.62–1.05 |
| **Chemotherapy Cycles** |  |  |  |  |
| < 4 | 52 | - | 1.00 | Reference |
| 4–6 | 360 | .04 | 0.61 | 0.38–0.99 |
| **TKI Targeted therapy** |  |  |  |  |
| None | 259 | - | 1.00 | Reference |
| Tarceva | 30 | .90 | 0.97 | 0.58–1.62 |
| Iressa | 98 | .03 | 0.71 | 0.52–0.97 |
| Conmana | 21 | .08 | 0.57 | 0.30–1.07 |
| Second Line TKI Osimertinib, Afatinib | 4^3^ | <.005 | 8.70 | 3.48–21.76 |
| **Moxa** |  |  |  |  |
| None | 117 | - | 1.00 | Reference |
| 1–4 times | 239 | .20 | 0.93 | 0.82–1.04 |
| > 4 times | 56 | .01 | 0.80 | 0.69–0.94 |

1. The IPTW-weighted Cox proportional-hazards regression analysis yielded results that were largely consistent with the unweighted model (Supplementary Table 6a), confirming the robustness of the findings. Significant predictors of survival: male sex, TNM Stage IVB, number of chemotherapy cycles, and Moxa frequency, remained stable across both models, with only minor variations in hazard ratios and P-values.
2. P-values reflect the probability of no significant difference, with the first category for each characteristic as the reference. Hazard ratios and 95% confidence intervals (CI) indicate the relative risk of death compared to the reference group. Although the unweighted model showed slightly better predictive accuracy (concordance index: 0.709 vs. 0.700 for IPTW), the consistency between models strengthens confidence in the observed associations between treatments and survival outcomes. This analysis performed using Python's statsmodels (v.0.14.3) and lifelines (v.0.29.0) libraries, supports the reliability of the results.
3. Results for categories with small patient numbers should be interpreted cautiously.

**Supplementary Table 6c.** Impact of baseline characteristics and treatments on survival: Cox proportional-hazards regression in stage IV adenocarcinoma patients in propensity score matched dataset (N = 230).

| **Characteristic/  Category** | **Total  (N = 230)^1^** | **P-value^2^** | **Hazard ratio** | **95% CI  of Hazard ratio** |
| --- | --- | --- | --- | --- |
| **Age (Years)** |  |  |  |  |
| 28–55 | 51 | - | 1.00 | Reference |
| 56–62 | 54 | .58 | 1.14 | 0.72–1.81 |
| 63–68 | 63 | .39 | 1.21 | 0.78–1.86 |
| 69–81 | 62 | .84 | 1.04 | 0.68–1.61 |
| **Sex** |  |  |  |  |
| Female | 123 | - | 1.00 | Reference |
| Male | 107 | .09 | 1.35 | 0.96–1.91 |
| **TNM Stage** |  |  |  |  |
| Stage IVA | 70 | - | 1.00 | Reference |
| Stage IVB | 160 | .0006 | 1.87 | 1.31–2.68 |
| **Smoking Habit Index** |  |  |  |  |
| No to mild | 188 | - | 1.00 | Reference |
| Heavy | 42 | .77 | 0.94 | 0.61–1.45 |
| **EGFR Mutations** |  |  |  |  |
| Absent | 205 | - | 1.00 | Reference |
| Present | 25 | .64 | 0.86 | 0.45–1.64 |
| **TCM Syndromes** |  |  |  |  |
| Deficiency of lung and spleen | 110 | - | 1.00 | Reference |
| Endogenous heat due to yin deficiency | 0 | - | - | - |
| Deficiency of qi and yin | 110 | .96 | 1.01 | 0.73–1.39 |
| Deficiency of spleen and kidney | 10 | .09 | 0.48 | 0.20–1.13 |
| **Radiotherapy** |  |  |  |  |
| No | 154 | - | 1.00 | Reference |
| Yes | 76 | .13 | 0.77 | 0.55–1.08 |
| **Chemotherapy Cycles** |  |  |  |  |
| < 4 | 26 | - | 1.00 | Reference |
| 4–6 | 204 | .35 | 0.75 | 0.42–1.36 |
| **TKI Targeted therapy** |  |  |  |  |
| None | 155 | - | 1.00 | Reference |
| Tarceva | 16 | .26 | 0.69 | 0.36–1.33 |
| Iressa | 44 | .03 | 0.58 | 0.36–0.94 |
| Conmana | 12 | .09 | 0.50 | 0.23–1.11 |
| Second Line TKI Osimertinib, Afatinib | 3^3^ | .01 | 5.86 | 1.63–21.06 |
| **Moxa** |  |  |  |  |
| None | 115 | - | 1.00 | Reference |
| 1–4 times | 96 | .01 | 0.65 | 0.47–0.90 |
| > 4 times | 19 | .0011 | 0.33 | 0.17–0.64 |

1. Propensity score matching improved balance but at the cost of reducing the sample size. As expected, the reduced dataset (from 412 to 230 patients) resulted in a slightly lower predictive ability (concordance index: 0.675 vs. 0.709).
2. Multivariate Cox proportional-hazards regression analysis using the propensity score-matched dataset (N = 230) in stage IV adenocarcinoma patients. P-values reflect the probability of no significant difference, with the first category for each characteristic serving as the reference. Hazard ratios and 95% confidence intervals (CI) indicate the relative risk of death compared to the reference group. Despite the reduced sample size, significant predictors of survival, such as TNM stage, Moxa frequency, and certain TKI therapies, were consistent with the results from the unweighted and IPTW analyses (Supplementary Tables 6a and 6b).
3. Results for categories with few observations should be interpreted with caution.

**Supplementary Table 7**. Comparison of the probabilities of no difference between the Kaplan-Meier survival curves (log-rank test), at different restricted mean follow-up durations of the None, 1–4, and > 4 Moxa frequency subgroups (N = 412) (RMST = restricted mean survival time). Note that the better survival rates in the different Moxa subgroups compared with no Moxa were significant over a long follow-up duration period.

| **Follow-up duration (month)** | **No Moxa**  **RMST (95% CI)** | **1–4 times Moxa**  **RMST (95% CI)** | **> 4 Moxa**  **RMST**  **(95% CI)** | **P-value**  **None  vs. Any^1^ (Log**  **Rank)** | **P-value  None vs. 1–4^2^ (Log**  **Rank)** | **P-value None vs. > 4^2^ (Log**  **Rank)** | **P-value 1–4 vs. > 4^2,3^**  **(Log**  **Rank)** |
| --- | --- | --- | --- | --- | --- | --- | --- |
|  | (n =117) | (N = 239) | (N = 56) |  |  |  |  |
| 12 | 10.9  (10.5–11.3) | 11.4  (11.1– 11.6) | 11.8  (11.5– 12.0) | .02 | .053 | .0002 | .01 |
| 18 | 14.7  (13.9– 15.5) | 15.9  (15.4– 16.4) | 17.2  (16.5– 17.8) | .003 | .02 | <.0001 | .002 |
| 24 | 17.9  (16.6– 19.2) | 19.69  (18.9– 20.5) | 22.2  (21.0– 23.3) | .002 | .02 | <.0001 | .0006 |
| 36 | 22.8  (20.6– 24.9) | 25.90  (24.5– 27.4) | 30.49  (28.20– 32.8) | .002 | .02 | <.0001 | .001 |
| 48 | 26.2  (23.3– 29.1) | 30.1  (28.1– 32.1) | 36.2  (32.6– 39.7) | .004 | .03 | <.0001 | .004 |
| 60 | 28.6  (25.0– 32.2) | 33.1  (30.5– 35.6) | 39.9  (35.1– 44.7) | .008 | .046 | .0002 | .01 |

1. Patients receiving Moxa >1 times had significantly better survival compared to those with no Moxa across all follow-up durations.
2. Patients receiving Moxa (“1–4 times” and “> 4 times”) had significantly better survival compared to those with no Moxa.
3. Survival benefits were more pronounced in the “> 4 times” group compared to “1–4 times” group, indicating a dose-dependent effect.

**Supplementary Table 8a.** Multivariate Kaplan-Meier survival analysis of Moxa and TKI-TT treatments with interaction terms, including log-rank test, hazard ratios, and 95% confidence intervals in stage IV adenocarcinoma patients (N = 412) with performance scores 0–1 and 3–120 months follow-up.

| **Moxa**  **and TKI-TT treatments**  **with interaction terms** | **Dead of disease**  **/at risk** | **% Censored (Alive with disease)** | **Median Survival Time (Months)** | **Probability of no difference (Log**  **Rank)** | **Hazard Ratio^1^**  **(95% CI)** |
| --- | --- | --- | --- | --- | --- |
| **Total^2^** | 323/412 | 22% | 30.7 |  |  |
|  |  |  |  |  |  |
| **Comparisons by Moxa (1–4 and > 4) and Any TKI-TT** | | | | | |
|  |  |  |  |  |  |
| ***Standard care:***  ***PBC + oral CHM only (Ref^3^)*** | 76/81^4,5^ | 6% | 20.0 |  |  |
|  |  |  |  |  |  |
| ***+ 1–4 Moxa*** | 118/151^6^ | 22% | 30.7 | .0053 | 0.68 (0.49–0.94) |
|  |  |  |  |  |  |
| ***+ TKI-TT*** | 28/36^7^ | 22% | 33.0 | .0114 | 0.56 (0.36–0.87) |
|  |  |  |  |  |  |
| ***+ TKI-TT  + 1–4 Moxa*** | 68/88^7^ | 23% | 33.0 | .0063 | 0.63 (0.44–0.90) |
|  |  |  |  |  |  |
| ***+ > 4 Moxa*** | 14/27^8^ | 48% | 32.0 | .0017 | 0.40 (0.24–0.65) |
|  |  |  |  |  |  |
| ***+ TKI-TT  + > 4 Moxa*** | 19/29^8^ | 34% | 40.0 | .0015 | 0.45 (0.29–0.72) |
|  |  |  |  |  |  |
| **Comparisons by Any Moxa and Any TKI-TT** | | | | | |
|  |  |  |  |  |  |
| ***Standard care:***  ***PBC + oral CHM only (Ref^3^)*** | 76/81^4,5^ | 6% | 20.0 |  |  |
|  |  |  |  |  |  |
| ***+ Any Moxa*** | 132/178^9^ | 26% | 32.0 | .0009 | 0.63 (0.46–0.89) |
|  |  |  |  |  |  |
| ***+ TKI-TT*** | 28/36^7^ | 22% | 33.0 | .0114 | 0.56 (0.36–0.87) |
|  |  |  |  |  |  |
| ***+ TKI-TT + Any Moxa*** | 87/117^10^ | 26% | 36.0 | .0005 | 0.58 (0.41–0.81) |

1. Hazard ratios of less than 1 indicate improved survival compared to standard care.
2. ***Total*** refers to the median survival for all patients in the study, regardless of their treatment group.
3. ***Ref:*** *Subgroup* Standard care: ***PBC + oral CHM only*** (N = 81) *is used as the reference group to calculate the significance, hazard ratios, and 95% CI calculations with the other subgroups.*
4. **None Moxa** in Table 1 (n =117) includes standard care (N = 81) and + TKI-TT (N = 36).
5. **None TKI-TT** in Table 1 (N = 259) includes standard care (N = 81), + 1–4 Moxa (N = 151), and + > 4 Moxa (N = 27).
6. **Moxa 1–4 times** in Table 1 (N = 239) includes + 1–4 Moxa (N = 151) and + TKI-TT + 1–4 Moxa (N = 88).
7. **Any TKI-TT** in Table 1 (N = 153) includes + TKI-TT (N = 36), + TKI-TT + 1–4 Moxa (N = 88), and + TKI-TT + > 4 Moxa (N = 29).
8. **Moxa > 4 times** in Table 1 (N = 56) includes + > 4 Moxa (N = 27) and + TKI-TT + > 4 Moxa (N = 29).
9. **Any Moxa** (N = 178) includes 1–4 Moxa (N = 151), and + > 4 Moxa (N = 27)
10. **TKI-TT + Any Moxa** (N = 117) includes TKI-TT + 1–4 Moxa (N = 88) and TKI-TT + > 4 Moxa (N = 29).

**Supplementary Table 8b.** Multivariate Kaplan-Meier survival analysis of Moxa and TKI-TT treatments with interaction terms, including log-rank test, hazard ratios, and 95% confidence intervals in stage IV adenocarcinoma patients (Propensity score matched dataset, N = 230) with performance scores at 0–1 and 3–120 months follow-up.

| **Moxa**  **and TKI-TT**  **with interaction terms** | **Dead of disease**  **/at risk** | **% Censored (Alive with disease)** | **Median Survival Time (Months)** | **Probability of no difference (Log**  **Rank)** | **Hazard Ratio^1^**  **(95% CI)** |
| --- | --- | --- | --- | --- | --- |
| **Total^2^** | 185/230 | 20% | 29.0 |  |  |
|  |  |  |  |  |  |
| **Comparisons by Moxa (1–4 and > 4) and Any TKI-TT** | | | | | |
|  |  |  |  |  |  |
| ***Standard care:***  ***PBC + oral CHM only (Ref^3^)*** | 76/80 | 5% | 19.0 |  |  |
|  |  |  |  |  |  |
| ***+ 1–4 Moxa*** | 47/63 | 25% | 37.0 | .0082 | 0.64 (0.43–0.94) |
|  |  |  |  |  |  |
| ***+ TKI-TT*** | 27/35 | 23% | 33.0 | .0099 | 0.56 (0.36–0.87) |
|  |  |  |  |  |  |
| ***+ TKI-TT  + 1–4 Moxa*** | 25/33 | 24% | 36.0 | .0053 | 0.53 (0.34–0.83) |
|  |  |  |  |  |  |
| ***+ > 4 Moxa*** | 6/12 | 50% | 47.0 | .0086 | 0.34 (0.18–0.63) |
|  |  |  |  |  |  |
| ***+ TKI-TT  + > 4 Moxa^6^*** | 4/7 | 43% | 41.1 | .0176 | 0.29 (0.15–0.59) |
|  |  |  |  |  |  |
| **Comparisons by Any Moxa and Any TKI-TT** | | | | | |
|  |  |  |  |  |  |
| ***Standard care:***  ***PBC + oral CHM only (Ref^3^)*** | 76/80 | 5% | 19.0 |  |  |
|  |  |  |  |  |  |
| ***+ Any Moxa*** | 53/75 | 29% | 37.0 | .0011 | 0.58 (0.40–0.84) |
|  |  |  |  |  |  |
| ***+ TKI-TT*** | 27/35 | 23% | 33.0 | .0099 | 0.56 (0.36–0.87) |
|  |  |  |  |  |  |
| ***+ TKI-TT + Any Moxa*** | 29/40 | 28% | 39.5 | .0007 | 0.48 (0.32–0.72) |

1. Hazard ratios of less than 1 indicate improved survival compared to the reference group.
2. **Total** refers to the median survival for all patients in the study, regardless of their treatment group. It represents the combined survival outcome for the propensity score matched cohort (N = 230), providing a general measure of how long patients survived on average, across all treatment groups.
3. **Ref:** Subgroup standard care: **PBC + oral CHM only** is used as the reference group to calculate the significance, hazard ratios, and 95% CI calculations with the other subgroups.

**Supplementary Figure 2.** Kaplan-Meier survival curves comparing standard care (PBC + oral CHM only), adding Moxa, TKI-TT, and a combination of those in stage IV adenocarcinoma patients (N = 412), indicating that frequent Moxa use, especially when combined with TKI-TT therapy, significantly improves long-term survival.


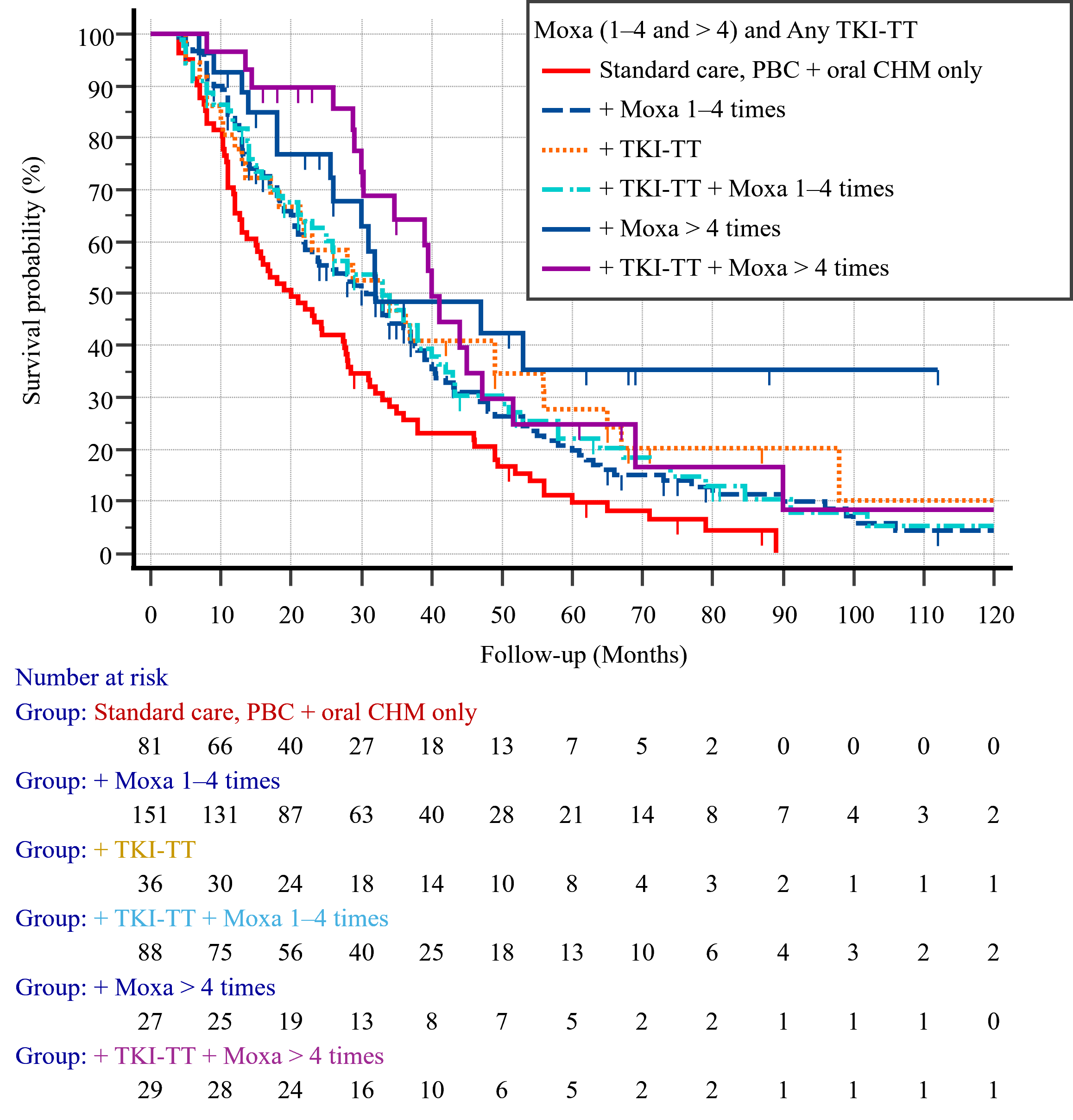


Note: The RMST analysis in Supplementary Tables 9a and 9b revealed that the survival benefit of > 4 Moxa treatments, in addition to TKI-TT, outperformed TKI-TT alone from 12 to 48 months in the full dataset, and from 24 to 60 months in the propensity score-matched dataset. The greatest survival benefit was observed in the “TKI-TT + > 4 Moxa” group.

**Supplementary Table 9a.** Comparison of survival outcomes across Moxa and TKI-TT treatment groups with interaction terms: mean survival, 95% confidence intervals, and P-values from restricted mean survival time (RMST) analysis at 12, 36, and 48 months (N = 412).

| **Follow-up time (months)** | **Moxa and TKI-TT treatments**  **with interaction terms** | **Total (N)** | **Mean**  **(months)** | **SE** | **95% CI for the Mean** | **P-value^2^ (RMST)** |
| --- | --- | --- | --- | --- | --- | --- |
| 12 | **Overall** | 412 | 11.29 | 0.09 | 11.12– 11.47 | - |
|  | Compared to standard care |  |  |  |  |  |
|  |  |  |  |  |  |  |
| 12 | ***Standard care:***  ***PBC + oral CHM only*** | 81 | 10.81 | 0.25 | 10.32– 11.30 | Reference category |
|  |  |  |  |  |  |  |
|  | ***+ 1–4 Moxa*** | 151 | 11.45 | 0.13 | 11.21– 11.70 | .0220 |
|  |  |  |  |  |  |  |
| 12 | ***+ TKI-TT*** | 36 | 11.15 | 0.32 | 10.51– 11.78 | .4143 |
| 12 | ***+ TKI-TT + 1–4 Moxa*** | 88 | 11.20 | 0.22 | 10.77– 11.63 | .2438 |
|  |  |  |  |  |  |  |
| 12 | ***+ > 4 Moxa*** | 27 | 11.70 | 0.21 | 11.29– 12.11 | .0067 |
| 12 | ***+ TKI-TT + > 4 Moxa*** | 29 | 11.86 | 0.14 | 11.60– 12.13 | .0002 |
|  | Compared to TKI-TT treatment |  |  |  |  |  |
|  |  |  |  |  |  |  |
| 12 | ***+ TKI-TT*** | 36 | 11.15 | 0.32 | 10.51– 11.78 | Reference category |
| 12 | ***+ TKI-TT + > 4 Moxa*** | 29 | 11.86 | 0.14 | 11.60– 12.13 | .0416 |
|  |  |  |  |  |  |  |
| 36 | **Overall** | 412 | 25.62 | 0.570 | 24.51– 26.72 | - |
|  | Compared to standard care |  |  |  |  |  |
|  |  |  |  |  |  |  |
| 36 | ***Standard care:***  ***PBC + oral CHM only*** | 81 | 21.45 | 1.300 | 18.92– 23.99 | Reference category |
|  |  |  |  |  |  |  |
| 36 | ***+ 1–4 Moxa*** | 151 | 25.73 | 0.920 | 23.92– 27.53 | .0073 |
| 36 | ***+ TKI-TT*** | 36 | 25.78 | 1.950 | 21.96– 29.60 | .0644 |
| 36 | ***+ TKI-TT + 1–4 Moxa*** | 88 | 26.15 | 1.240 | 23.73– 28.57 | .0087 |
|  |  |  |  |  |  |  |
| 36 | ***+ > 4 Moxa*** | 27 | 28.88 | 1.850 | 25.26– 32.50 | .0010 |
| 36 | ***+ TKI-TT + > 4 Moxa*** | 29 | 31.96 | 1.410 | 29.21– 34.72 | <.0001 |
|  | Compared to TKI-TT treatment |  |  |  |  |  |
|  |  |  |  |  |  |  |
| 36 | ***+ TKI-TT*** | 36 | 25.78 | 1.950 | 21.96– 29.60 | Reference category |
| 36 | ***+ TKI-TT + > 4 Moxa*** | 29 | 31.96 | 1.410 | 29.21– 34.72 | .0101 |
|  |  |  |  |  |  |  |
| 48 | ***Overall*** | 412 | 29.79 | 0.79 | 28.25– 31.34 | - |
|  | Compared to standard care |  |  |  |  |  |
|  |  |  |  |  |  |  |
| 48 | ***Standard care:***  ***PBC + oral CHM only*** | 81 | 24.22 | 1.73 | 20.84– 27.61 | Reference category |
|  |  |  |  |  |  |  |
| 48 | ***+ 1–4 Moxa*** | 151 | 29.86 | 1.30 | 27.31– 32.41 | .0091 |
| 48 | ***+ TKI-TT*** | 36 | 30.72 | 2.76 | 25.32– 36.12 | .0458 |
| 48 | ***+ TKI-TT + 1–4 Moxa*** | 88 | 30.42 | 1.71 | 27.08– 33.76 | .0107 |
| 48 | ***+ > 4 Moxa*** | 27 | 34.63 | 2.90 | 28.95– 40.31 | .0020 |
| 48 | ***+ TKI-TT + > 4 Moxa*** | 29 | 37.68 | 2.21 | 33.35– 42.01 | <.0001 |
|  | Compared to TKI-TT treatment |  |  |  |  |  |
|  |  |  |  |  |  |  |
| 48 | ***+ TKI-TT*** | 36 | 30.72 | 2.76 | 25.32– 36.12 | Reference category |
| 48 | ***+ TKI-TT + > 4 Moxa*** | 29 | 37.68 | 2.21 | 33.35– 42.01 | .0485 |

1. Moxa and TKI-TT combinations significantly improved survival compared to standard care. The greatest survival benefit was observed in the +TKI-TT + > 4 Moxa group, with RMST analysis showing notable survival improvements compared to TKI-TT alone.

**Supplementary Table 9b.** Comparison of survival outcomes across Moxa and TKI-TT treatment groups with interaction terms: mean survival, 95% confidence intervals, and P-values from restricted mean survival time (RMST) analysis at 24, 36, 48, and 60 months (N = 230).

| **Follow-up time (months)** | **Moxa**  **and TKI-TT treatments**  **with interaction terms** | **Total (N)** | **Mean**  **(months)** | **SE** | **95% CI for the Mean** | **P-value^1,2^ (RMST)** |
| --- | --- | --- | --- | --- | --- | --- |
| 24 | **Overall** | 230 | 19.01 | 0.44 | 18.14– 19.87 | - |
|  | Compared to standard care |  |  |  |  |  |
|  |  |  |  |  |  |  |
| 24 | ***Standard care:***  ***PBC + oral CHM only*** | 80 | 17.18 | 0.80 | 15.61– 18.75 | Reference category |
|  |  |  |  |  |  |  |
|  | ***+ 1–4 Moxa*** | 63 | 19.26 | 0.82 | 17.65– 20.87 | .0690 |
| 24 | ***+ TKI-TT*** | 35 | 19.24 | 1.14 | 17.01– 21.46 | .1383 |
| 24 | ***+ TKI-TT + 1–4 Moxa*** | 33 | 20.53 | 1.01 | 18.54– 22.52 | .0094 |
|  |  |  |  |  |  |  |
| 24 | ***+ > 4 Moxa*** | 12 | 22.03 | 1.42 | 19.25– 24.80 | .0024 |
| 24 | ***+ TKI-TT + > 4 Moxa*** | 7 | 24.00 | 0.00 | 24.00– 24.00 | <.0001 |
|  |  |  |  |  |  |  |
|  | Compared to TKI-TT treatment |  |  |  |  |  |
|  |  |  |  |  |  |  |
| 24 | ***+ TKI-TT*** | 35 | 19.24 | 1.14 | 17.01– 21.46 | Reference category |
| 24 | ***+ TKI-TT + > 4 Moxa*** | 7 | 24.00 | 0.00 | 24.00– 24.00 | .0416 |
|  |  |  |  |  |  |  |
| 36 | **Overall** | 230 | 24.95 | 0.78 | 23.43– 26.47 | - |
|  | Compared to standard care |  |  |  |  |  |
|  |  |  |  |  |  |  |
| 36 | ***Standard care:***  ***PBC + oral CHM only*** | 80 | 21.28 | 1.3 | 18.73– 23.83 | Reference category |
| 36 | ***+ 1–4 Moxa*** | 63 | 25.77 | 1.51 | 22.82– 28.72 | .00241 |
| 36 | ***+ TKI-TT*** | 35 | 25.49 | 1.98 | 21.60– 29.37 | .00759 |
| 36 | ***+ TKI-TT + 1–4 Moxa*** | 33 | 27.73 | 1.89 | 24.04– 31.43 | .0048 |
|  |  |  |  |  |  |  |
| 36 | ***+ > 4 Moxa*** | 12 | 30.14 | 2.63 | 24.99– 35.28 | .0025 |
| 36 | ***+ TKI-TT + > 4 Moxa*** | 7 | 35.78 | 0.2 | 35.40– 36.17 | <.0001 |
|  |  |  |  |  |  |  |
|  | Compared to TKI-TT treatment |  |  |  |  |  |
|  |  |  |  |  |  |  |
| 36 | ***+ TKI-TT*** | 35 | 25.49 | 1.98 | 21.60– 29.37 | Reference category |
| 36 | ***+ TKI-TT + > 4 Moxa*** | 7 | 35.78 | 0.2 | 35.40– 36.17 | <.0001 |
|  |  |  |  |  |  |  |
| 48 | ***Overall*** | 230 | 29.21 | 1.08 | 27.09– 31.33 | - |
|  | Compared to standard care |  |  |  |  |  |
|  |  |  |  |  |  |  |
| 48 | ***Standard care:***  ***PBC + oral CHM only*** | 80 | 23.98 | 1.73 | 20.60– 27.37 | Reference category |
|  |  |  |  |  |  |  |
| 48 | ***+ 1–4 Moxa*** | 63 | 30.48 | 2.12 | 26.34– 34.63 | .00174 |
| 48 | ***+ TKI-TT*** | 35 | 30.55 | 2.83 | 25.00– 36.10 | .04477 |
| 48 | ***+ TKI-TT + 1–4 Moxa*** | 33 | 32.95 | 2.72 | 27.62– 38.27 | .0054 |
|  |  |  |  |  |  |  |
| 48 | ***+ > 4 Moxa*** | 12 | 36.63 | 4.1 | 28.60– 44.66 | .0049 |
| 48 | ***+ TKI-TT + > 4 Moxa*** | 7 | 42.55 | 1.93 | 38.76– 46.34 | <.0001 |
|  |  |  |  |  |  |  |
|  | Compared to TKI-TT treatment |  |  |  |  |  |
|  |  |  |  |  |  |  |
| 48 | ***+ TKI-TT*** | 35 | 30.55 | 2.83 | 25.00– 36.10 | Reference category |
| 48 | ***+ TKI-TT + > 4 Moxa*** | 7 | 42.55 | 1.93 | 38.76– 46.34 | .0005 |
|  |  |  |  |  |  |  |
| 60 | ***Overall*** | 230 | 32.34 | 1.36 | 29.68– 34.99 | - |
|  | Compared to standard care |  |  |  |  |  |
|  |  |  |  |  |  |  |
| 60 | ***Standard care:***  ***PBC + oral CHM only*** | 80 | 25.68 | 2.05 | 21.66– 29.69 | Reference category |
|  |  |  |  |  |  |  |
| 60 | ***+ 1–4 Moxa*** | 63 | 33.95 | 2.69 | 28.68– 39.22 | .00143 |
| 60 | ***+ TKI-TT*** | 35 | 34.6 | 3.6 | 27.54– 41.66 | .0313 |
| 60 | ***+ TKI-TT + 1–4 Moxa*** | 33 | 37.34 | 3.58 | 30.32– 44.36 | .0047 |
|  |  |  |  |  |  |  |
| 60 | ***+ > 4 Moxa*** | 12 | 41.91 | 5.57 | 30.99– 52.83 | .0063 |
| 60 | ***+ TKI-TT + > 4 Moxa*** | 7 | 46.55 | 4.04 | 38.63– 54.47 | <.0001 |
|  |  |  |  |  |  |  |
|  | Compared to TKI-TT treatment |  |  |  |  |  |
|  |  |  |  |  |  |  |
| 60 | ***+ TKI-TT*** | 35 | 34.6 | 3.6 | 27.54– 41.66 | Reference category |
| 60 | ***+ TKI-TT + > 4 Moxa*** | 7 | 46.55 | 4.04 | 38.63– 54.47 | .00273 |

1. Moxa combined with TKI-TT treatments significantly improved survival compared to standard care at all follow-up points (24, 36, 48, and 60 months). The greatest survival benefit was observed in the +TKI-TT + > 4 Moxa group, with highly significant results. RMST analysis showed that Moxa combined with TKI-TT consistently outperformed TKI-TT alone. The +1–4 Moxa group also showed survival benefits, with higher frequencies of Moxa leading to greater improvements.
2. This analysis on the propensity score-matched dataset confirms findings from the full dataset, where RMST revealed that > 4 Moxa treatments, in addition to TKI-TT, outperformed TKI-TT alone from 12 to 48 months and in the matched dataset from 24 to 60 months.
