# Supplementary figures and images for: Moxibustion Treatment, Alongside Conventional Western and Chinese Herbal Medical Therapies, May Improve Survival in Stage-IV Pulmonary Adenocarcinomas in a Dosage-Dependent Manner: A Prospective Observational Study With Propensity Score Analysis
Source: Integr Cancer Ther. 2025 Jun 19;24:15347354251342739. doi: 10.1177/15347354251342739 (PMC12179449; doi:10.1177/15347354251342739)

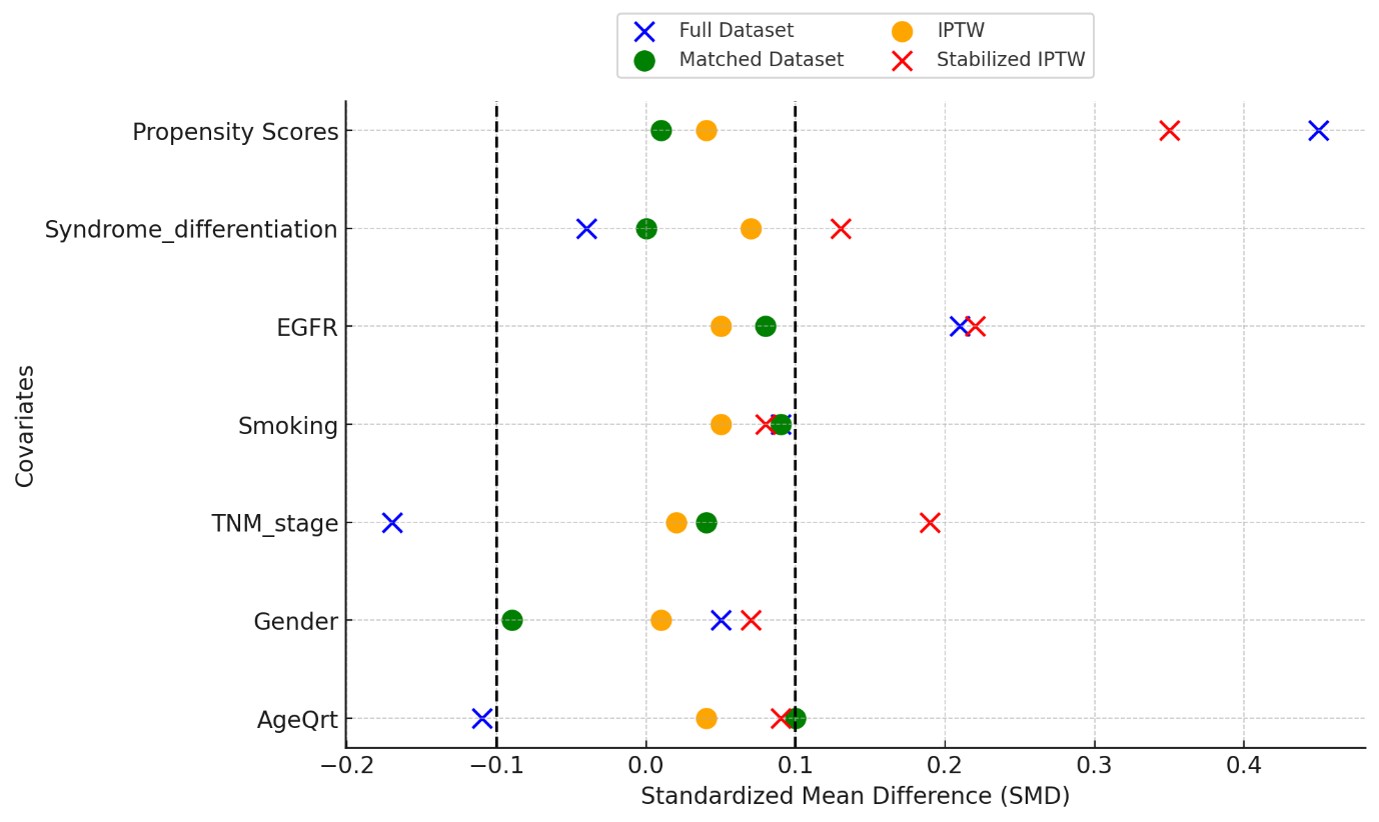

Supplement: sj-jpg-1-ict-10.1177_15347354251342739 – Supplemental material for Moxibustion Treatment, Alongside Conventional Western and Chinese Herbal Medical Therapies, May Improve Survival in Stage-IV Pulmonary Adenocarcinomas in a Dosage-Dependent Manner: A Prospective Observational Study With Propensity Sc [file sj-jpg-1-ict-10.1177_15347354251342739.jpg]

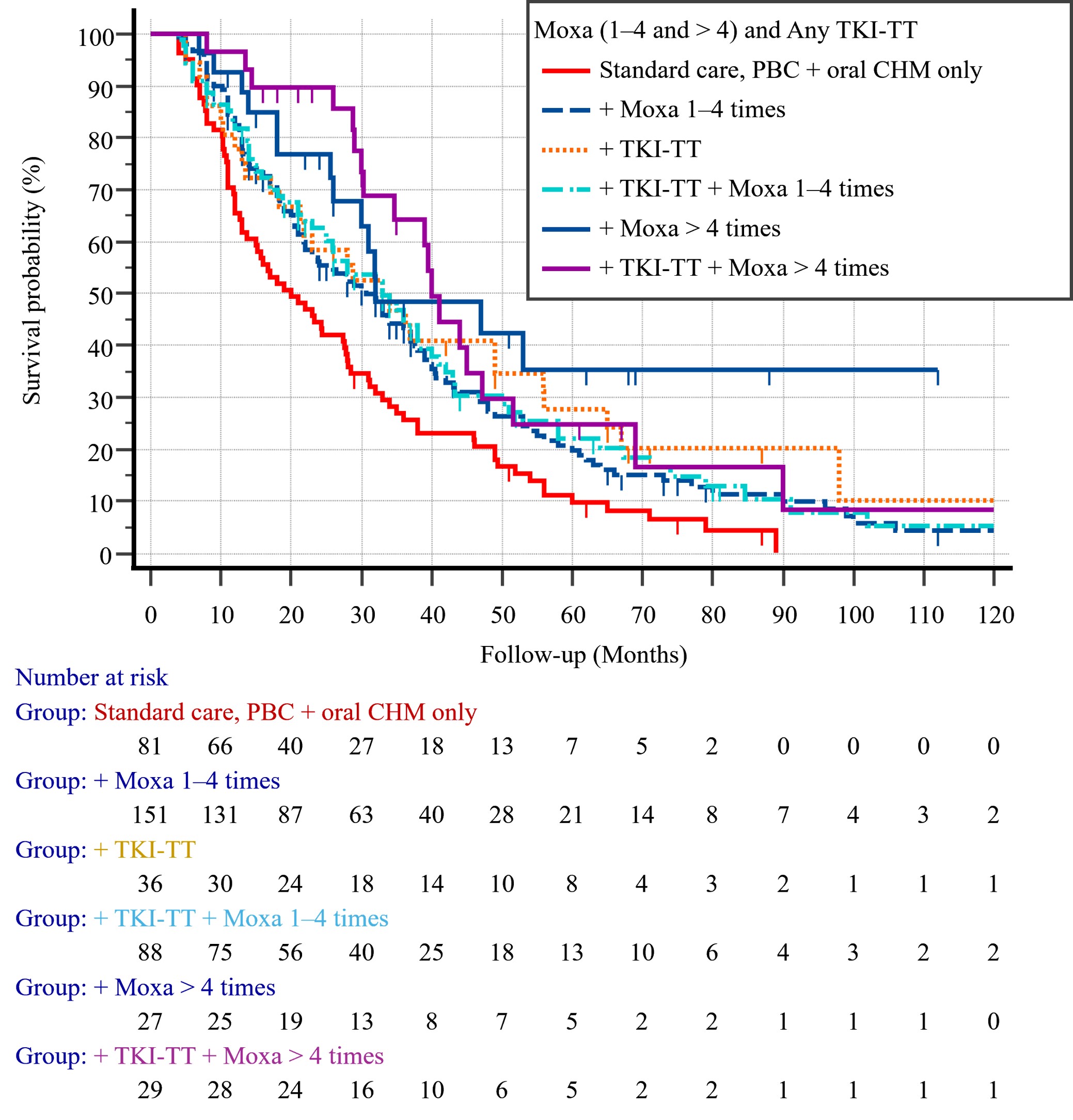

Supplement: sj-jpg-2-ict-10.1177_15347354251342739 – Supplemental material for Moxibustion Treatment, Alongside Conventional Western and Chinese Herbal Medical Therapies, May Improve Survival in Stage-IV Pulmonary Adenocarcinomas in a Dosage-Dependent Manner: A Prospective Observational Study With Propensity Sc [file sj-jpg-2-ict-10.1177_15347354251342739.jpg]
